# Analytic Element Domain Boundary Conditions for Site-Scale Groundwater Flow Modeling Los Angeles Basin

**DOI:** 10.1111/gwat.13322

**Published:** 2023-05-04

**Authors:** Stephen R. Kraemer

**Affiliations:** 1 U.S. Environmental Protection Agency, Office of Research and Development, 75 Hawthorne St, San Francisco, CA 94105, USA

## Abstract

Physics-based groundwater flow modeling is a useful tool for the design and optimization of pump-and-treat systems for groundwater site cleanup. Numerical methods like finite differences and finite elements, and hybrid analytic elements, require boundary conditions (BC) to be assigned to the outer domain of the grid, mesh, or line elements. These outer BC do not always correspond with hydrogeologic features. Common practice in model setup is to either: (1) extend the model domain boundary outward such that introduced artificial outer BCs (e.g., first type head specified, second type flux specified) do not have undue influence on near-field scale simulations; or (2) assign outer BCs to capture the effective far-field influence (e.g., third type head-dependent flux). Groundwater flow modeling options for assigning BCs were demonstrated for the extensively documented Dual Site Superfund cleanup in Torrance, California. The existing MODFLOW models for the Dual Site scale and the Los Angeles basin scale document the current hydrogeologic conceptual site model. Simplified analytic element AnAqSim models at the LA Basin scale, West Coast Subbasin scale, and Dual Site scale, were used for mapping near-field domain velocity vector fields and pathline envelopes. The pump-treat-inject system demonstrated hydraulic containment and showed pathline envelopes relatively insensitive to BC choices. However, the nearfield domain boundary groundwater flow fields were sensitive to BC choices. The Los Angeles basin case study demonstrated the use of analytic element groundwater modeling for testing stress dependent boundaries during site pump-treat-inject design.

## Introduction

Computer modeling is useful for the design and optimization of pump-and-treat (P&T) groundwater cleanup systems (EPA 1997, 1999a; Matott et al. 2006; Compernolle et al. 2013). Modeling is an important tool in helping to test and evolve the conceptual site model (CSM) that integrates geology and contaminant transport mechanisms (Kresic and Mikszewski 2013) and for P&T system design (Truex et al. 2017). Sensitivity analysis reveals the importance of boundary conditions (BC) used in computational flow modeling on the design of P&T systems (Matott 2012).

### Hydrogeological Conceptual Site Model and Boundary Conditions

An important component of a computational model application to site-specific problems is the assignment of outer domain BC (Franke et al. 1984; Reilly 2001; ASTM 2016). The mathematical expressions for Dirichlet specified head (Type 1), Neumann specified flux (Type 2), and Robin head-dependent flux (Type 3) are reported by Jazayeri and Werner (2019) and summarized in Table S1.

The definition of BC is part of the application of a groundwater model to a site-specific problem (ASTM 2016). The steps are described as: (1) identification of the physical boundaries of the groundwater flow system boundaries; (2) formulation of the mathematical representation of the boundaries; (3) review of sensitivity testing of boundary conditions that change when the system is under stress, that is, stress dependent boundaries; (4) revision of the formulation of the initial model boundary representation; and (5) further evaluation, testing, and refinement of the model boundaries as a part of the verification and validation process of the application of each model.

If the boundary conditions are stress dependent, the model cannot be considered a general, all-purpose tool for investigating any stress on the system because it will give valid results only when the stresses do not impact the boundary (ASTM 2016 6.4.1.2).

Good modeling practice involves the defensible assignment of outer domain boundary conditions with particular attention given when model boundaries differ from physical boundaries of the natural groundwater flow system. Ideally, all model boundaries would correspond with hydrogeological boundaries, that is, radiating outward from the area of interest, or site scale, a physical boundary is included, with the assignment of specified head or specified flux based on field observation. With closed domain simulations, this is rarely practical, or necessary. Common practice in conceptual model setup is to either: (1) extend the model near-field domain boundary such that introduced artificial outer BCs do not unduly influence the site scale simulations; or (2) to assign outer BCs to the site scale to capture the effective far-field influence under expected site stress scenarios (e.g., Type 3 head-dependent flux). The goal to extend the modeling domain to minimize outer BC impact is often countered by the goal of focusing computational power (high grid resolution) at the plume scale for contaminant transport simulations.

Most computational models, such as those involving finite-difference or finite-element solutions, require setting some artificial boundary conditions on outer cells of a grid or mesh. An exception is the analytical element method (Strack 1989; Haitjema 1995) where the mathematical elements are superimposed within an infinite spatial domain and far-field influences may be set at a reference point. The representation of Dirichlet head specified BC and Neumann flux specified BC using finite-difference methods are described in Reilly (2001). The analytic element model AnAqSim (Fitts 2010) allows the building of hybrid analytic element models with closed external domain BCs and head specified (Dirichlet, Type 1) and normal flux specified (Neumann, Type 2). The practical use of the Robin head-dependent flux (Type 3) BC is implemented in MODFLOW as a general head boundary (GHB) (Figure S1a) and in AnAqSim as line elements (Figure S1b).

**Figure 1.**
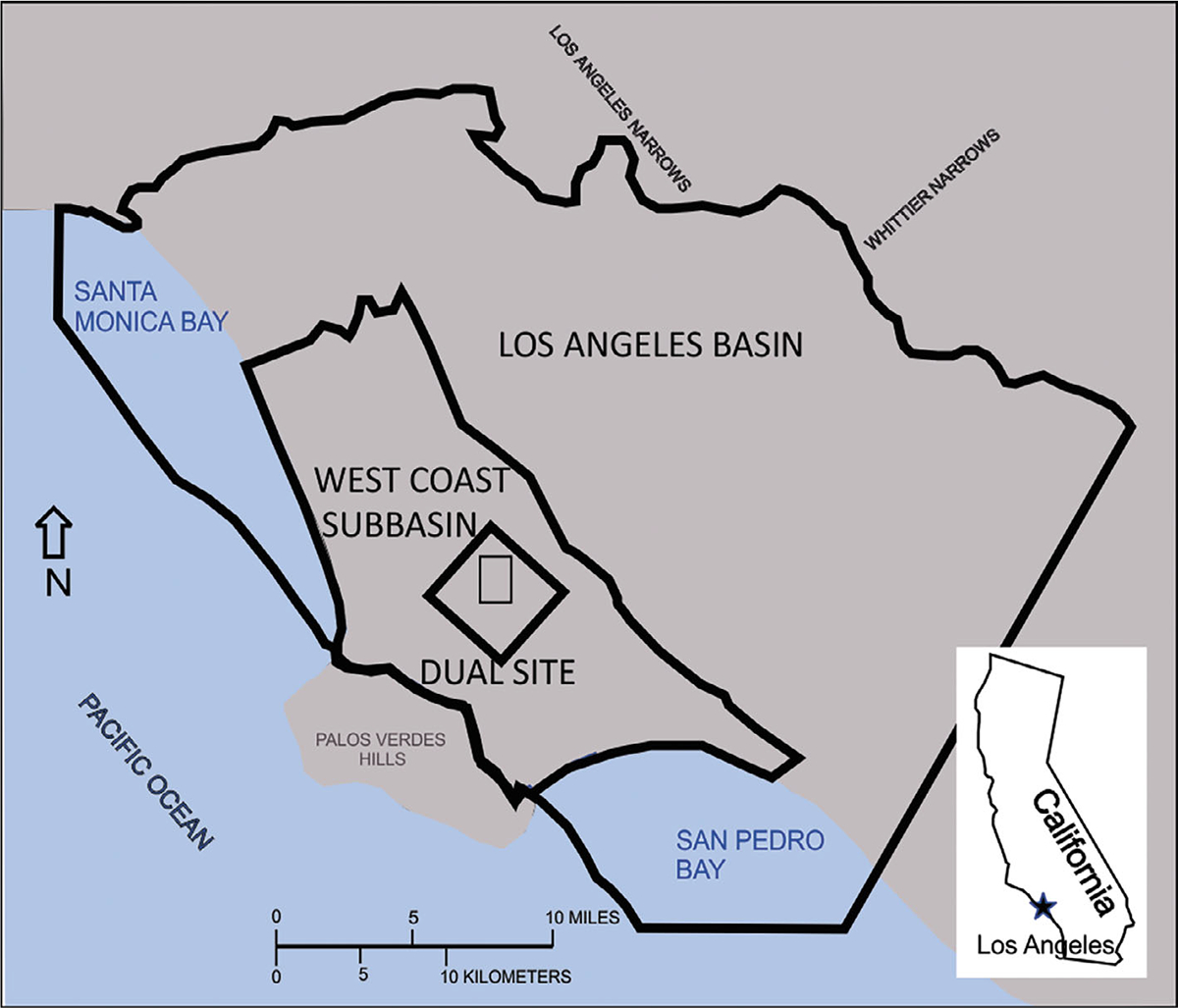
The three domains for this investigation of groundwater flow modeling and boundary conditions as stressed by the pump-treat-inject system of the Montrose-Del Amo Superfund cleanup: (1) the Los Angeles Basin; (2) the West Coast Subbasin; and (3) the Dual Site.

The well documented hydrogeologic CSM for the mature and ongoing cleanup of the Dual Site Superfund operable unit in the Los Angeles basin provides an opportunity for a retrospective sensitivity analysis of outer BC choices given the stress fields created by the proposed pump-treat-inject system. I demonstrate a deterministic modeling approach using the analytic element method with special attention given to the choice of conditions defining the outer boundary of the simulation domain at three spatial scales: (1) Dual Site scale; (2) Los Angeles Basin scale; and (3) West Coast Subbasin scale (Figure 1). The model generated maps of near-field domain velocity vector fields and pathline envelopes for the wells provided the basis for comparison and discussion.

### The Dual Site Case Study

The groundwater site characterization and modeling at the Montrose-Del Amo Superfund Site has been proceeding over decades. The analysis presented in this paper is informed by existing public information, is for general insights, and is not part of the site-specific investigation and groundwater cleanup.

Montrose Chemical Corporation of California (Montrose) operated as a chemical manufacturing plant on 13 acres in the city of Torrance from 1947 to 1982, when it was closed, disassembled, and removed. The neighboring Del Amo site is 280 acres located in a narrow strip in the Harbor Gateway near Torrance and included a synthetic rubber plant from 1943 to 1972.

The Dual Site Groundwater Operable Unit at the Montrose-Del Amo Superfund sites has a co-mingled dissolved plume associated with plumes from multiple sources, including dense nonaqueous phase liquids (DNAPLs)—DDT (dichloro-diphenyl-trichloroethane), PCE (perchloroethylene), TCE (trichloroethylene), para-chlorobenzene sulfonic acid (pCBSA); and light nonaqueous phase liquids (LNAPLs)—benzene.

The Superfund workflow has included a National Priorities listing (NPL), the Remedial Investigation/Feasibility Study (RI/FS) (EPA 1998; CH2M Hill 1998), the Record of Decision (ROD) (EPA 1999b, 2019), and the current phase of Remedial Actions. The ROD requires hydraulic isolation of nonaqueous phase liquid (NAPL) and dissolved phase contamination surrounding the NAPL in a containment zone, and restoration of the contaminated groundwater outside the containment zone to an in situ groundwater standard (EPA 1999b).

The expected behavior of the plumes in the hidden subsurface is captured in the hydrogeological CSM. The DNAPL will tend to move vertically (downward) and horizontally in a cascade manner in the presence of finite tight lenses in the stratified sand aquifer layers. The dissolved plumes will tend to move horizontally in the presence of an advective groundwater flow system. The stratigraphy is described in the ROD and includes a layercake geometry from top to bottom.

**Figure 2.**
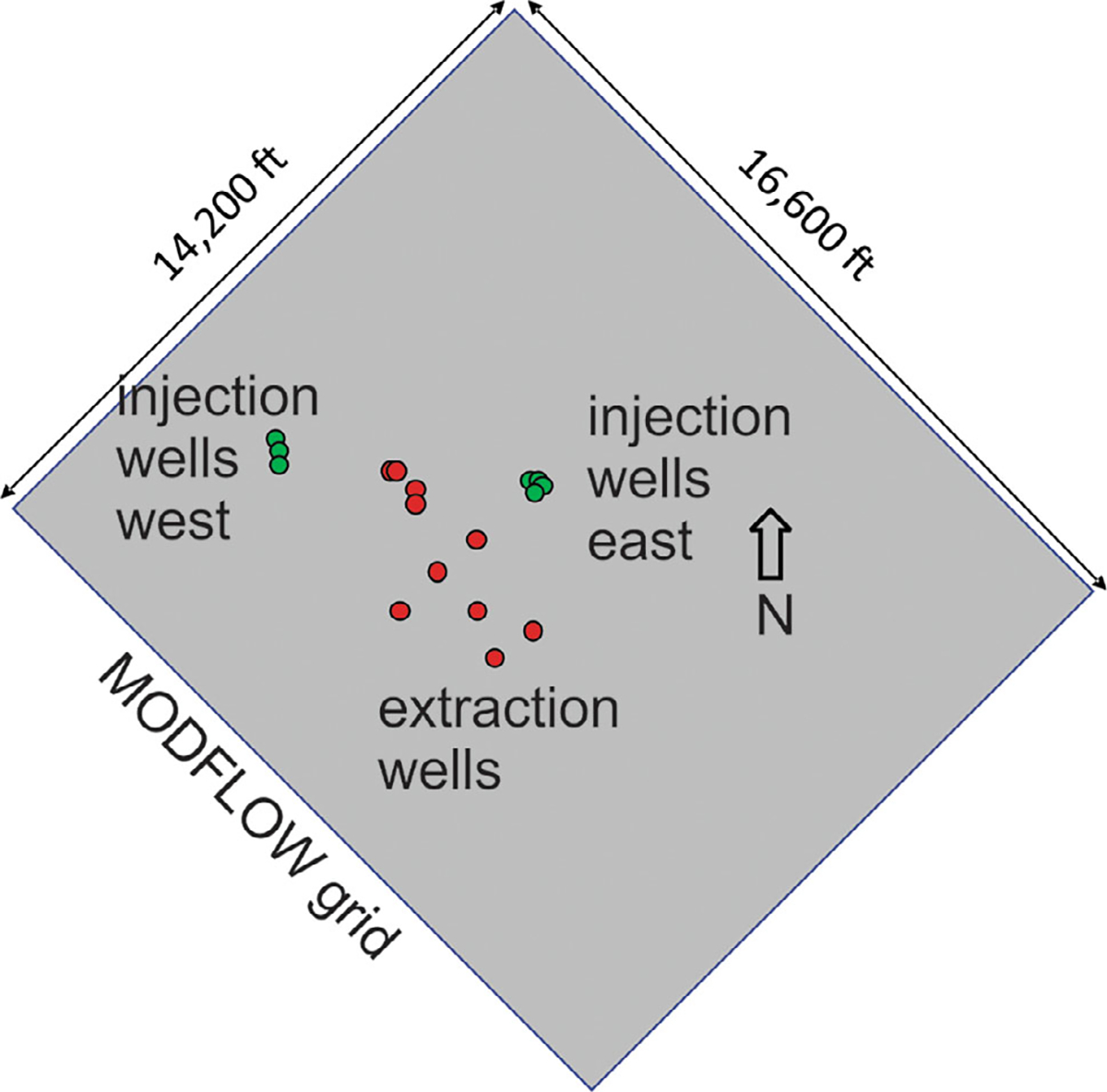
The layout of the Dual Site groundwater cleanup system (pump-treat-inject) within the MODFLOW grid.

The RI/FS documents the treatability testing and evaluation of the performance and cost of treatment technologies. The ROD describes the selected response action as the treatment of the chlorobenzene plume using hydraulic extraction (pumping), treatment, aquifer reinjection, at a design total rate of approximately 700 gallons per minute (gpm), assuming a closed water balance system, with 410 gpm western injection and 290 gpm eastern injection (EPA 2019) (Figure 2). The Dual Site MODFLOW model being used in the planning has 166 rows, 126 columns, and 13 layers and covers an area 14,200 feet (4328.2 m) by 16,600 feet (5059.7 m) (CH2M Hill 2008) (Figure 2). The treatment system presented here includes 7 injection wells and 11 extraction wells.

**Figure 3.**
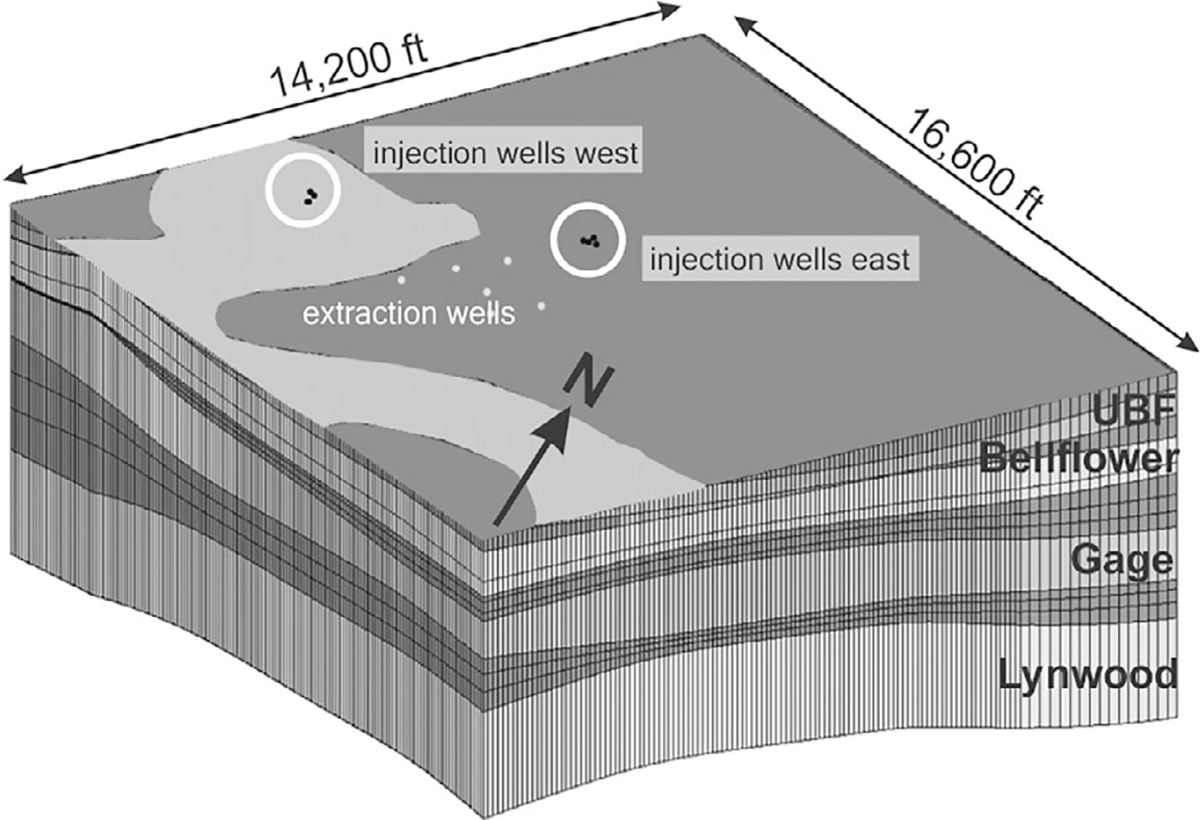
MODFLOW hydrostratigraphic units (HSUs) described by average hydraulic conductivity (ft/d) and bottom elevation (feet above mean sea level). The aquitards (darker: Upper Bellflower aquitard (1.8 ft/d; −41.2 feet), Middle Bellflower (27.3 ft/d; −49.9 feet) M (9. 6ft/d; −57.7 feet) aquitard, Lower Bellflower aquitard (0.075 ft/d; −118.2 feet), Gage-Lynwood aquitard (0.021 ft/d; −223.3 feet). The aquifers (lighter): Bellflower Sand (58.0 ft/d; −93.6 feet), Gage (31.5 ft/d; −177.5 feet), Lynwood (54.2 ft/d; −323.2 feet). Vertical exaggeration is 20×.

While the geology is heterogeneous and varied, the layers appear effectively horizontal, as visualized with the MODFLOW grid (Figure 3).

A combination of general head (GHB) and no-flow boundaries were used in the contractor generated Dual Site MODFLOW model (CH2M Hill 1998, 2008). For each GHB cell, a reference head was assigned outside the model domain, and the conductance parameterized to reproduce the interpreted heads at the model boundary. The interpreted heads came from the contour maps associated with the historical record of water levels in wells. The MODFLOW model was calibrated to steady state conditions for October 2006 synoptic observations of heads (CH2M Hill 2008) as summarized in Figure S2 (Gage Aquifer Layer 9 mean residual heads +0.01 feet [4.17E−3 m], normalized root mean square error [RMSE] 5.9%). The MODPATH pathlines associated with the pump-treat-inject system are visualized based on reverse particle tracking from the extraction wells in the Gage and other aquifers (Figure S3) suggesting a closed pathline envelope from injection wells to extraction wells in the Gage aquifer. The following analytic element modeling explores hypothetical scenarios and the testing of pathline envelopes based on the pump-treat-inject system and BC at basin, subbasin, and site scales.

## Methods

Existing and published MODFLOW finite-difference models provided the foundation and context for the study of the sensitivity of BCs at multiple scales as stressed by the proposed pump-treat-inject system. I translated the MODFLOW information into the analytic element model AnAqSim (www.FittsGeoSolutions.com; Fitts 2010) with the overall spatial structure of nested domains and levels. The analytic element method is based on the superposition of point-sinks (wells), line-sinks (rivers, interfaces), linedipoles (drains, fractures), and polygons (line elements bounding and separating domains) (https://en.wikipedia.org/wiki/Analytic_element_method). Since the purpose of this modeling study was for comparative insight, and not site-specific optimization, a manual model calibration strategy with the goal of randomly distributed minimized residual heads (average model minus observed heads less then plus/minus 2ft/0.61 m) and normalized RMSE less than 20% was deemed acceptable for the simplified representations.

### Dual Site Scale Model Layout

**Figure 4.**
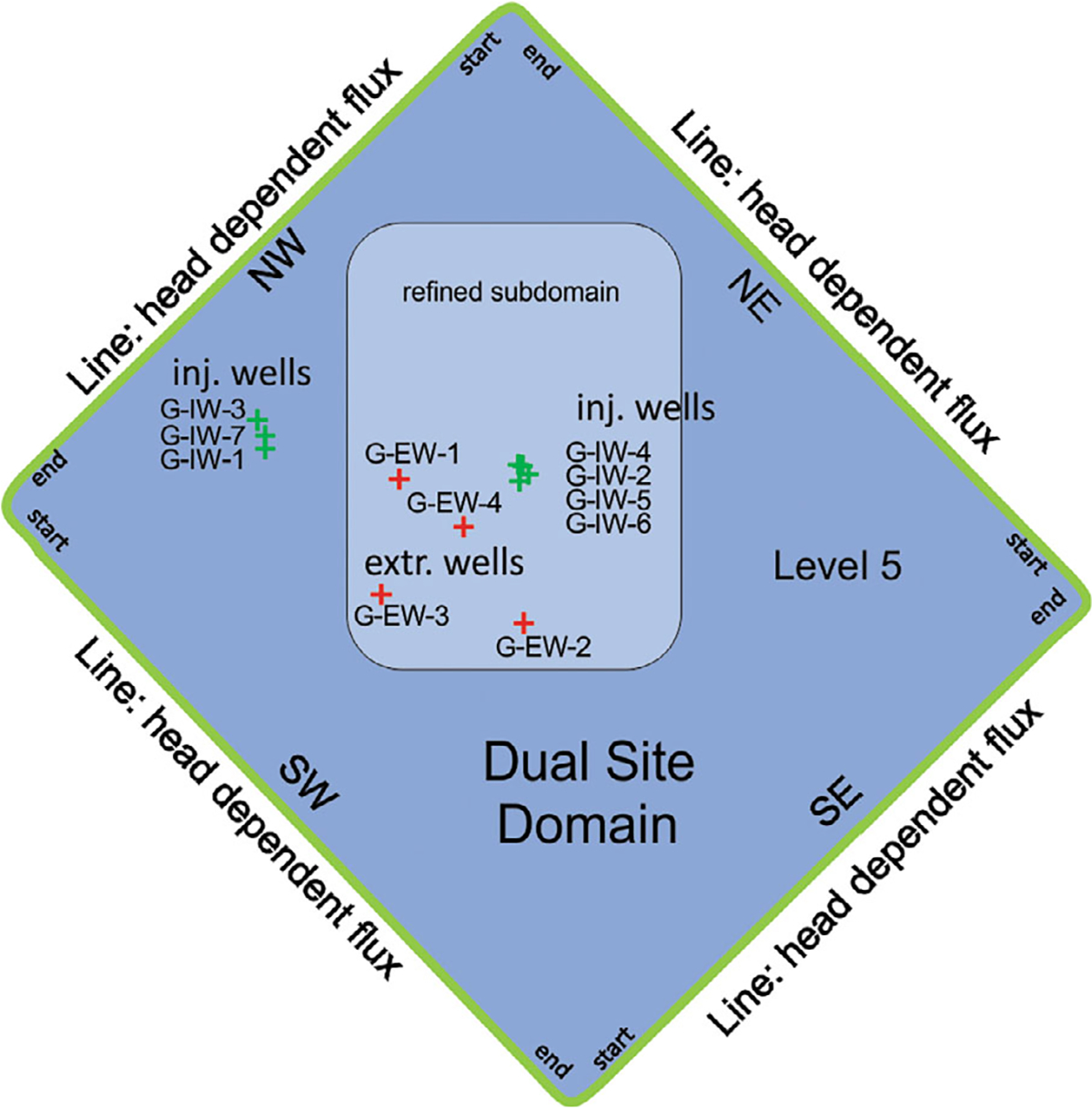
AnAqSim layout of elements for the Dual Site domain. The domain has five levels. The Gage aquifer is labeled level 5. The domain is surrounded by linear head-dependent flux (Type 3) BC and parameterized by conductance (1/*d*) and heads (feet) interpolated between start and end. The refined subdomain has 10 levels. The Gage injection and extraction wells are shown.

The Dual Site AnAqSim model was parameterized with data associated with the Dual Site MODFLOW model (CH2M Hill 1998). The 13-layer MODFLOW model of cell-by-cell variable layer elevations and properties was translated into the multidomain horizontal levels AnAqSim model for the Dual Site (Figure 4 and Table S2). The Dual Site domain was simplified to five AnAqSim horizontal levels (from shallow to deep, Mesa, Pacific, Bent Spring, Upper Wilmington, and Lower Wilmington). The embedded subdomain was reserved for an environmental sequence stratigraphy (Shultz et al. 2017) under development by ICF/Jacobs (2023, personal communication) and assigned 10 AnAqSim horizontal levels (from shallow to deep, Overburden, Upper Bellflower aquitard, Middle Bellflower aquifer, Lower Bellflower aquitard, Gage Aquifer, Gage-Lynwood aquitard, Upper Lynwood aquifer, Lower Lynwood aquifer, Upper Wilmington, Lower Wilmington). The MODFLOW cell-by-cell horizontal hydraulic conductivities (HK) by layer were averaged into AnAqSim isotropic horizontal hydraulic conductivity associated with levels (K1 = K2). The layered MODFLOW cell-by-cell vertical hydraulic conductivity (VK) were captured in AnAqSim level top half and bottom half hydraulic conductivity (K3) and anisotropy ratios ranged from 1 of 5 to 1 of 100, mostly 1 to 10, of horizontal hydraulic conductivity K1, K2, and domains were given a porosity of 0.3 (Table S2). The pump-treat-inject system (700 gpm) and wells were translated into AnAqSim with 7 injection wells in the Gage aquifer level 5, and 11 extraction wells, with 4 in the Gage aquifer level 5, and the rest in the Bellflower aquitard and aquifer (Table S3).

AnAqSim handles the Robin head-dependent flux third type BC with linear elements defining the outer domain of a level (Figure S1b). The GHB conductance of the MODFLOW cells were translated into the AnAqSim line boundaries by dividing the MODFLOW conductance (ft^3^/d) by the AnAqSim cross-sectional area (constant thickness times length) (ft^2^) to give the AnAqSim conductance (1/*d*). The MODFLOW GHB far-field head was used as the AnAqSim hypothetical head (h*) outside the boundary and a linear interpolation between heads at the start and end nodes (Table S4).

### LA Basin Scale Model Layout

**Figure 5.**
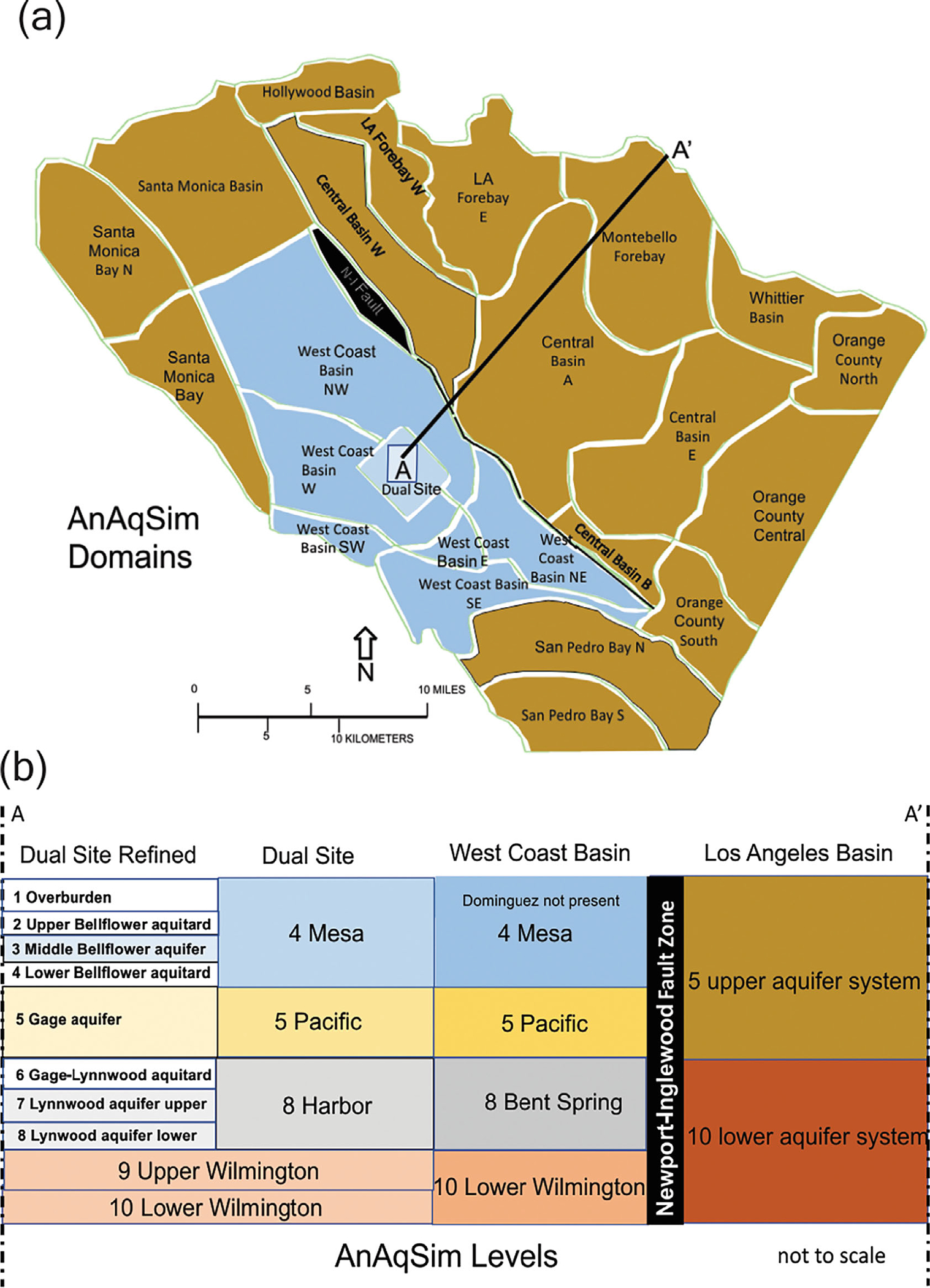
AnAqSim model representation of the LA Basin with: (a) domains, shown for level 5; and (b) levels, generalized cross-section informed by LA Basin lithostratigraphy, West Coast Basin and Dual Site chronostratigraphy.

The AnAqSim LA Basin model includes the Dual Site model domain and extends to the far-field basin scale BCs as represented by the U.S. Geological Survey (USGS) MODFLOW models (Figure 5 and Table S5). The domains of the LA Basin were divided into two levels in AnAqSim informed by the lithostratigraphy and hydrogeology reported by Reichard et al. (2003): an upper Pleistocene Lakewood Formation (Level 5) and a lower Pleistocene San Pedro Formation (Level 10). The domains of the West Coast Subbasin were divided into four levels in AnAqSim informed by the chronostratigraphy and hydrogeology of Paulinski (2021). The Dual Site scale domains and levels were previously described.

**Figure 6.**
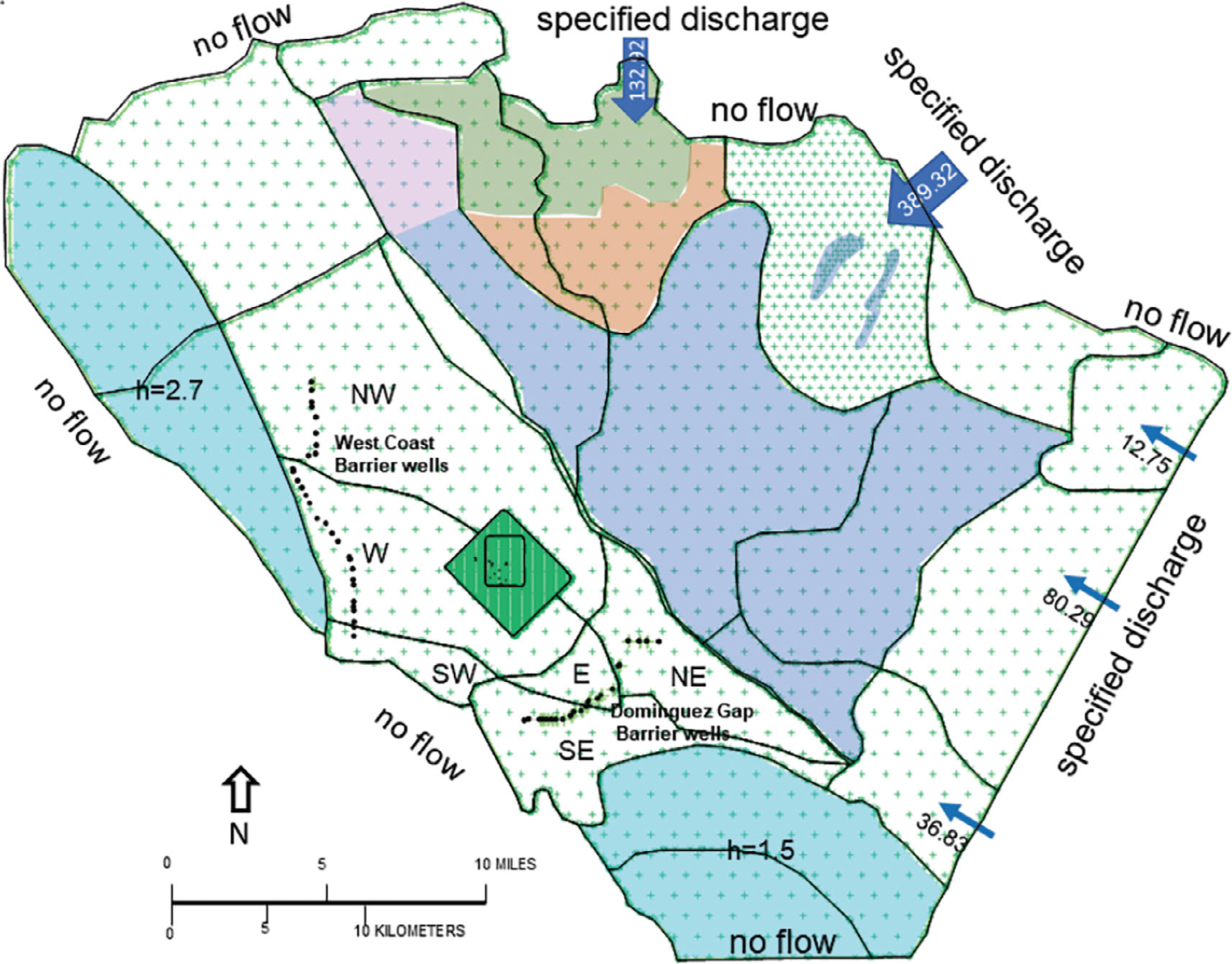
The AqAqSim LA Basin scale boundary conditions (BC). Spatially variable area sources/sinks (SVAS) represented a top-level flux over an area, or net recharge/exfiltration (ft/d)—the color shaded SVAS are defined by polygons that do not necessarily follow the boundaries of single domains. Positive flux for infiltration; negative flux for exfiltration. The bay areas are assigned a freshwater equivalent head top condition. The West Coast Basin areas left for assignment after calibration. The density of spacing of basis points ranges from 400 feet (site scale domains) to 4000 feet (basin scale domains). The Montebello Forebay spreading grounds handled with separate SVAS polygons. Normal flux line BC include no-flow and discharges per length (ft^2^/d) along Orange County and Los Angeles and Whittier Narrows.

The average annual water balance (1971 to 2015) for subbasins of the LA Basin MODFLOW reported in Paulinski (2021) was translated into AnAqSim elements: spatially variable area source/sinks (SVAS), and normal flux specified lines (Figure 6). The USGS net recharge (acre-ft/yr) (infiltration minus pumpage), was translated into SVAS top level area flux (ft/d), by polygon or by domain (Tables S6 and S7). The domains associated with the Santa Monica Bay and San Pedro Bay were given constant top level heads. The boundary labeled Orange County was represented with AnAqSim normal discharge line elements from Paulinski (2021); the location and geometry of the artificial BC (e.g., political boundary not associated with a hydrogeological feature) was corroborated through simulations with the single layer steady GFLOW model (Haitjema 1995) of the entire Los Angeles basin (Los Angeles and Orange Counties) as shown in Figure S4 through S6. The Los Angeles Narrows and Whittier Narrows groundwater flows were translated from USGS MODFLOW groundwater flow (acre-ft/yr) (Paulinski 2021) into AnAqSim normal flux specified line elements (ft^2^/d). The surrounding hills and outer ocean boundaries were represented with AnAqSim normal discharge (no-flow) line elements.

### West Coast Subbasin Scale Model Layout

**Figure 7.**
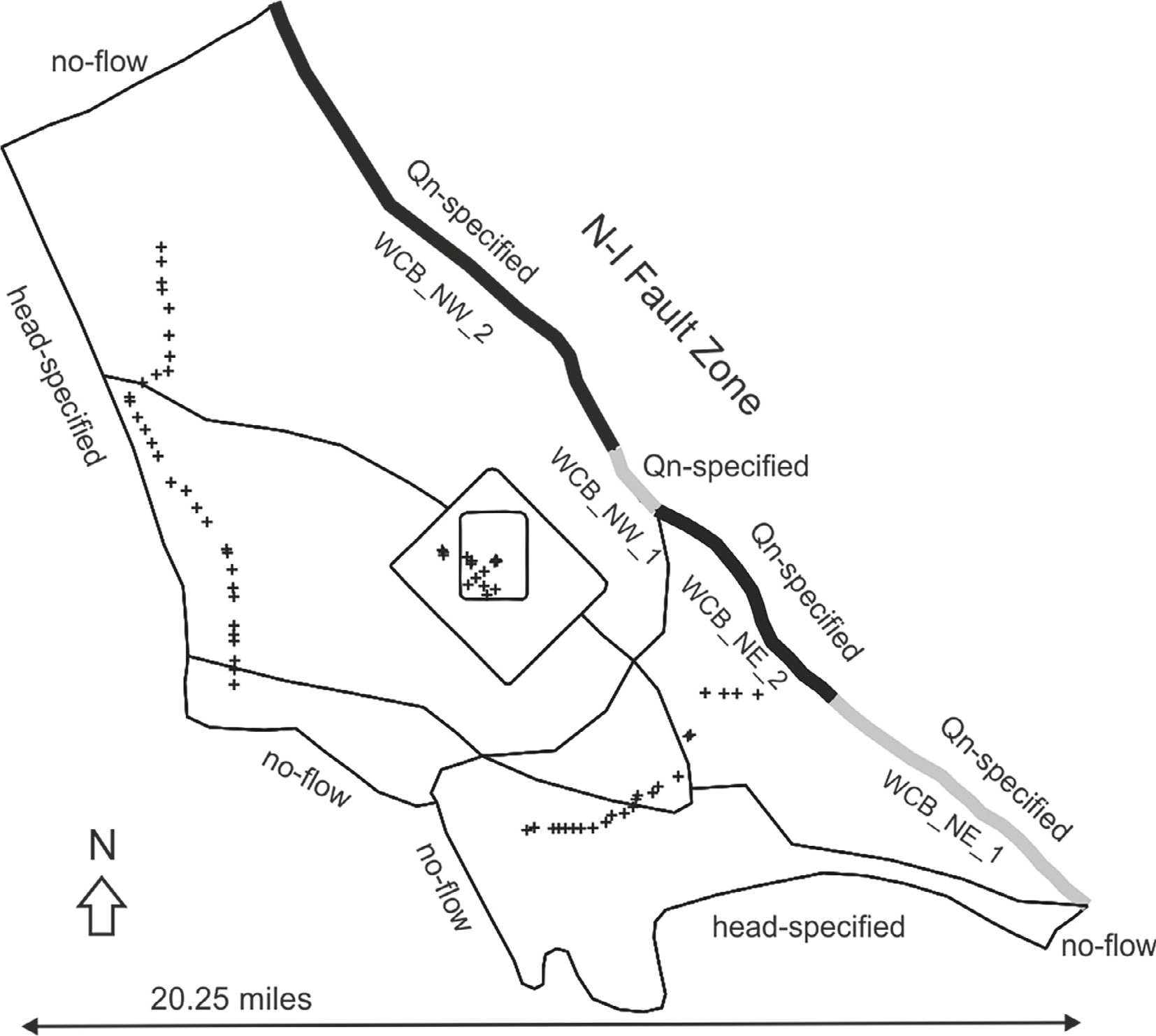
The AnAqSim model of the West Coast Subbasin. All domains and levels extracted from the AnAqSim LA Basin scale model, although the N-I Fault domain boundary is represented with normal flux specified line elements. The layout includes elements from all levels.

The AnAqSim model representing the West Coast Subbasin (Figure 7) was informed by the USGS Los Angeles MODFLOW model (Paulinski 2021) and the AnAqSim LA Basin Scale model. The BCs include specified flux (no flow) for the north-west Ballona Creek BC and the south-east Orange County as far-field features. A no-flow BC was also used to represent the Palos Verdes Hills to the south. The Newport-Inglewood (NI) Fault zone was parameterized with normal flux specified line segments during calibration as discussed in the results section. The Santa Monica Bay and San Pedro Bay boundaries were assigned specified-head line elements all levels (density adjusted freshwater heads of 2.7 feet) (0.823 m) and 1.5 feet (0.457 m), respectively. The recharge rates for the West Coast Subbasins were not explicitly fixed but reserved as calibration variables. The injections from the West Coast and Dominguez Gap seawater intrusion barrier wells were based on Paulinski (2021, Figure C5b) average rates for the decade 2010 and explicitly included in the AnAqSim model.

## Results

### Dual Site Scale Model Solution

The Dual Site AnAqSim model was calibrated to the observed hydraulic heads in Gage aquifer wells, averaged for the month October 2006 (CH2M Hill 2008) (Figure S7). I manually adjusted the recharge over the domain areas—AnAqSim spatially variable area sink (SVAS) for the Dual Site domain and the refined subdomain—and a value of 2.9E−4 ft/d (8.84E−5 m/d) yielded a minimized average residual for the Gage aquifer: the mean residual head is −0.028 feet (−8.53E-3 m) and the normalized RMSE is 10.04%.

**Figure 8.**
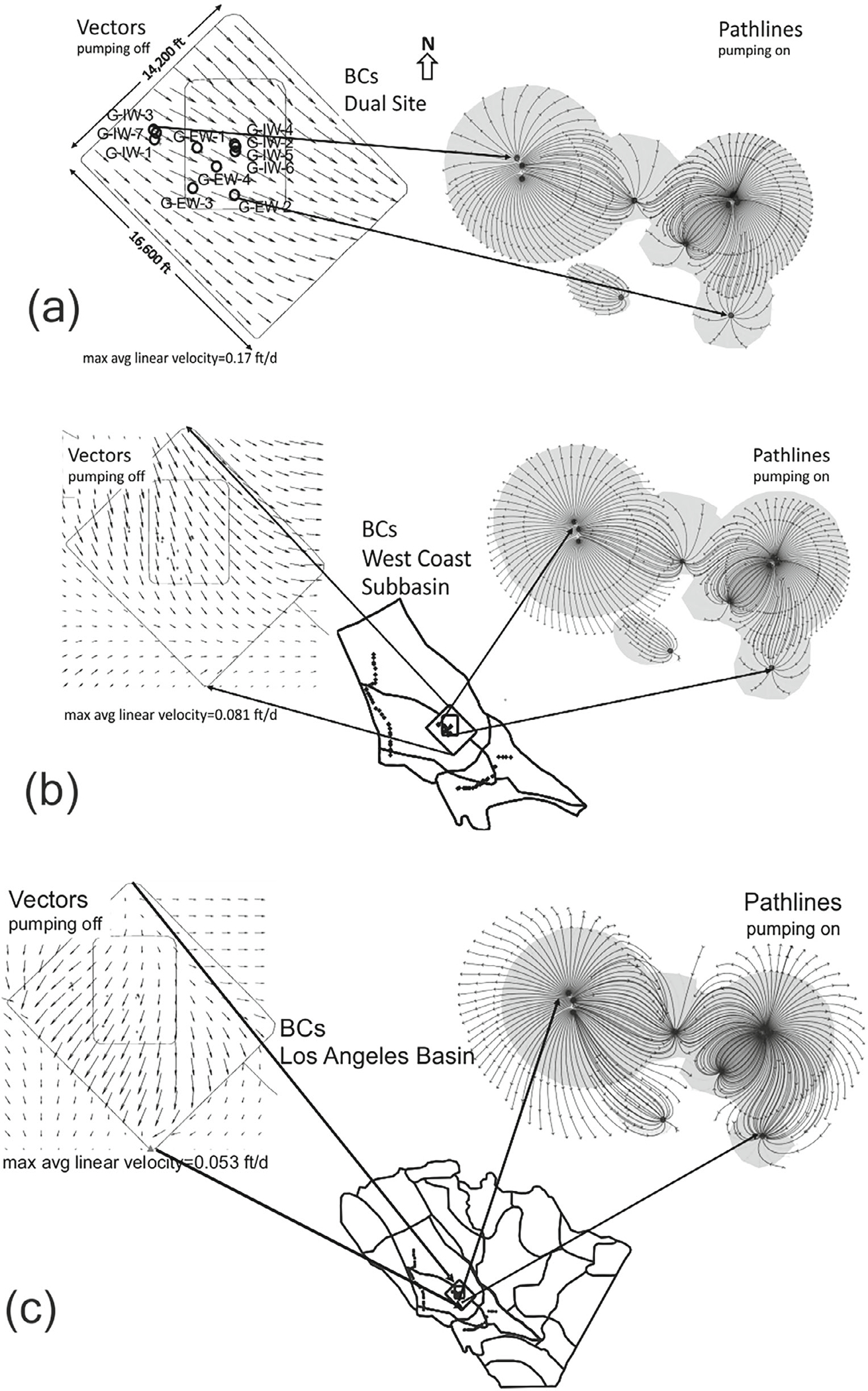
Comparison of AnAqSim solutions for nonpumping velocity vectors and injection/pumping pathlines with15 year residence time and 5 year tic marks, for (a) the Dual Site scale; (b) the West Coast Subbasin scale; and (c) the Los Angeles Basin scale. The injection-pumping pathline envelope for the Dual Site scale is shaded and added to the other pathline plots to assist in the comparison.

The pump-extract wells were added to the AnAqSim Dual Site scale model and the vector flow field (pumping off) and the pathline plot (pumping on) for Level 5 (Gage aquifer) are shown in Figure 8a. The velocity vector field suggest a relatively uniform flow to the south-east with maximum average linear velocity of 0.17 ft/d (0.052 m/d). The residence time associated with the pathline traces leaving the injection wells or entering the pumping wells was limited to 15years, with tic marks every 5 years. The projected life of the pump-treat-inject system is 15 years (CH2M Hill 2008). The pump-treat-inject area of influence is shaded to assist comparison with the other scenarios.

### LA Basin Scale Model Solution

The LA Basin scale AnAqSim model was calibrated to the observed hydraulic heads of the Gage Aquifer in the West Coast Subbasin (Figure S8). The Gage aquifer water levels were recorded in USGS wells for various dates in 2006 and archived by the Southern California Water Replenishment District (WRD 2007). I performed a simplified manual calibration by varying the exfiltration due to pumping within the West Coast Basin domain. A minimized average residual of −1.4 feet (0.43 m) and normalized RMSE of 18.8% was achieved with the exfiltration rate of −8E−4 ft/d (−2.44E−4 m/d) using the AnAqSim SVAS elements for the West Coast Basin (W, E, and SW domains).

The AnAqSim hydraulic head contour map from the calibration shows the drawdown in the Central Basin due to overdraft. The historical condition of overdraft motivated the drilling of the series of freshwater injection wells to manage seawater intrusion; the West Coast Barrier wells and the Dominguez Gap Barrier wells are included in the AnAqSim model (Figure 6).

The pump-treat-extract wells were added to the AnAqSim LA Basin scale model. The area of influence of the pump-treat-inject wells was superimposed as a shaded area of drawdown contours, plus or minus 0.05 feet (0.015 m), for the Gage aquifer (Figure S8a). The velocity vector field of pathline tracings for Level 5 (Gage aquifer) are mapped for the Dual Site area of the AnAqSim LA Basin model (Figure 8c). The vector field suggests nonuniform direction with maximum average linear velocity of 0.053 ft/d (0.016 m/d). The 15 year residence time pathline traces for the injection and pumping wells define quite a bit larger area than the Dual Site pathline trace (shaded area).

### West Coast Subbasin Scale Model Solution

The West Coast Subbasin scale AnAqSim model was calibrated to the WRD observations for heads in the Gage aquifer for 2006 (WRD 2007), the same head test points as the LA Basin scale AnAqSim model. I performed manual calibration by adjusting select AnAqSim elements to minimize the difference between observation and model prediction at test points (observed heads). The WCB parameterization that minimized the residuals involved the normal specified fluxes (positive removes water from the subbasin) of the N-I fault zone (WCB_NE_1 = 2 ft^2^/d, WCB_NE_2 = 4 ft^2^/d, WCB_NW_1 = 2 ft^2^/d, NW_2 = 2ft^2^/d), and the spatially variable area sources (SVAS) (WCB NW = 4.0E−4 ft/d, WCB W = −1E−03 ft/d, WCB E = −1E−03 ft/d, WCB SE = 1.0E−04 ft/d). The contours of head with test point residuals, scatter plot, and the statistics are reported in Figure S9. The West Coast Subbasin scale model had minimized average residual heads of −0.39 ft (−0.12 m) and normalized RMSE of 19.2%.

The pump-treat-inject wells were added to the AnAqSim West Coast Subbasin model and plots were made of the velocity vector field and pathline traces for Level 5 (Gage aquifer) (Figure 8b). The vector field suggests a nonuniform direction with maximum average linear velocity of 0.081 ft/d (0.0247 m/d). The pathline envelopes are in-between the size of the Dual Site and LA Basin envelopes.

## Discussion

The retrospective study of the pump-treat-inject system of the Dual Site Los Angeles demonstrates modeling concepts related to stress testing the near-field and far-field BCs. The case study was chosen based on the maturity of the field data and the availability of finite-difference groundwater flow MODFLOW models at site scales (contractor) and basin scales (USGS). The Dual Site analytic element model (AnAqSim) was parameterized using the Dual Site MODFLOW data positioning the tool for BC pump-treat-inject stress testing. The mapping of the Level 5 (Gage aquifer) pathline envelopes with 15 year residence times for the pump-treat-inject system were presented for comparisons at basin and subbasin scales.

The results show a consistent mapping of the residence time envelopes for the three scales (site, subbasin, and basin). It was not surprising that a pump-treat-inject system with zero net flow optimized for hydraulic containment (700 gpm) would be relatively insensitive to the influence of outer domain BCs. What was surprising was the variability of groundwater velocity vectors at the Dual Site modeling boundary for the basin-scale and subbasin-scale solutions prior to the initiation of the pump-treat-inject system. The AnAqSim Dual Site modeling calibrated to site observations of synoptic heads (October 2006) in the Gage aquifer suggests a uniform flow (max. avg. linear velocity 0.17 ft/d [0.0518 m/d]) to the SE direction (Figure 8a). Compare the WRD maps of hydraulic heads for the West Coast Basin and the Central Basin in the deeper aquifers for 2006 suggesting uniform flow in the Dual Site area of interest in the NE direction (Figure S10). AnAqSim modeling including West Coast Subbasin BC weakly suggests that the Dual Site area might be under nonuniform flow (max. avg. linear velocity 0.081 ft/d [0.0247 m/d]) in the Gage aquifer (Figure 8b). And AnAqSim modeling reaching out to LA Basin BC suggests a Gage aquifer water divide in the Dual Site area of interest (max. avg. linear velocity 0.053 ft/d). The AnAqSim models shared a similar manual calibration process, with the West Coast Subbasin and LA Basin having the same test point targets (observed heads Gage aquifer 2006), but different degrees of freedom and element types. In the absence of sufficient field observations of hydraulic heads to parameterize Dual Site scale modeling with head-dependent flux (Type 3) outer BC, the analysis suggests that extending the modeling domain to the West Coast Subbasin scale would be justified. The modeling underscores that the field monitoring campaign in the West Coast Subbasin would benefit from multiple synoptic surveys (wet season, dry season) and a multilayer perspective. In this mode of modeling practice, the simulations are intended to help guide the priorities for future field data collection (Haitjema 1995, section 5.3.2). This would be especially important in the early design phase of the pump-treat-inject system where the number of wells, location of wells, and hydraulic containment is being optimized.

In many practical cases, site scale groundwater cleanups are performed in basins that have peer-reviewed regional geologic conceptual models and calibrated numerical aquifer flow models. Interested parties engaged in remedial investigation and feasibility study are encouraged to take advantage of the existing groundwater modeling infrastructure and knowledge base when designing a stepwise site-scale data collection and groundwater modeling effort that includes stress dependency testing of outer BC choices.

## Conclusion

The retrospective case study involving the analytic element groundwater flow model AnAqSim and the well documented Dual Site cleanup in the Los Angeles Coastal Plain explored physics-based modeling practice in formulating geohydrologic conceptual models and assigning boundary conditions (BC) for the purpose of hydraulic containment pump-treat-inject design. The existing Dual Site MODFLOW model (166 rows, 126 columns, 13 layers; over 188,000 active cells of resolution 100 feet by 100 feet) and Basin Scale MODFLOW model (256 rows, 312 columns, 13 layers, over 440,000 cells of resolution 660 feet by 660 feet) provided the current and accepted geohydrologic conceptual site model. Extensive observations of hydraulic head and pumping tests allowed calibration of head-dependent flux outer domain BC of the Dual Site MODFLOW model. The simplified AnAqSim models at the LA Basin scale (24,503 equations) and West Coast Subbasin scales (16,289 equations) and Dual Site scale (13,013 equations) proved useful for stress dependency testing of outer domain BC associated with hydrogeologic features. The resulting mapping of velocity vector fields and pathline envelopes provided insights. The closed-water balance of the pump-treat-inject system made the pathline envelopes relatively insensitive to BC choices. However, the nearfield domain boundary flow fields were sensitive to BC choices. The efficiency of the analytic element method makes it well positioned for guiding early design phase evolution of the geohydrologic conceptual site model, and assisting in setting priorities for field data collection and defensible BC.

## Supplementary Material

Supplement1

## Article impact statement:

Analytic element model demonstrates impact of boundary conditions choices for pump-treatinject system in retrospective case study Los Angeles basin.
