# Supplementary material for: Analytic Element Domain Boundary Conditions for Site-Scale Groundwater Flow Modeling Los Angeles Basin
Source: Ground Water. Author manuscript; Available in PMC 2024 Sep 1. (PMC10546891; doi:10.1111/gwat.13322)
Supplement: Supplement1 [file NIHMS1930600-supplement-Supplement1.pdf]

Case Study/

Analytic Element Domain Boundary Conditions for Site-Scale Groundwater Flow Modeling, Los Angeles Basin

Stephen R. Kraemer
Corresponding author: U.S. Environmental Protection Agency, Office of Research and Development, 75 Hawthorne St, San Francisco, CA 94105; kraemer.stephen@epa.gov

Supporting Information

Abstract

Physics-based groundwater flow modeling is a useful tool for the design and optimization of pump and treat systems for groundwater site cleanup. Numerical methods like finite differences and finite elements, and hybrid analytic elements, require boundary conditions (BC) to be assigned to the outer domain of the grid, mesh, or line elements. These outer BC do not always correspond with hydrogeologic features. Common practice in model setup is to either: (1) extend the model domain boundary outward such that introduced artificial outer BCs (e.g. first type head specified, second type flux specified) do not have undue influence on near-field scale simulations; or (2) assign outer BCs to capture the effective far-field influence (e.g. third type head-dependent flux). Groundwater flow modeling options for assigning BCs were demonstrated for the extensively documented Dual Site Superfund cleanup in Torrance, California. The existing MODFLOW models for the Dual Site scale and the Los Angeles basin scale document the current hydrogeologic conceptual site model. Simplified analytic element AnAqSim models at the LA basin scale, West Coast subbasin scale, and Dual Site scale, were used for mapping near-field domain velocity vector fields and pathline envelopes. The pump-treat-inject system demonstrated hydraulic containment and showed pathline envelopes relatively insensitive to BC choices. However, the near-field domain boundary groundwater flow fields were sensitive to BC choices. The Los Angeles basin case study demonstrated the use of analytic element groundwater modeling for testing stress dependent boundaries during site pump-treat-inject design.

Disclaimer: Supporting Information is *not* peer reviewed.

Groundwater flow model BCs are classified as three main types: (Type 1) Dirichlet specified head including constant head; (Type 2) Neumann specified flux including no-flow; and (Type 3) Robin head dependent flux. Jazayeri and Werner (2019) frame the boundary problem for groundwater flow systems within a closed region Ω with boundary Γ and $\boldsymbol{x}$ representing temporal or spatial dimensions, and ϕ (representing hydraulic head) and n (representing the normal direction) and a (representing a non-zero coefficient). BCs are referenced by three main types as summarized in **Table S1**.

Table S1. Groundwater model boundary condition types (BC) after Jazayeri, Werner (2019).

Name BC	Descriptors	Mathematical Expression
Dirichlet (**Type 1**)	Specified head, including constant head	$\phi = f(\boldsymbol{x});\ \boldsymbol{x} \in \Gamma$
Neumann (**Type 2**)	Specified flux, including no-flow	$\frac{\partial \phi}{\partial n} = f(\boldsymbol{x});\ \boldsymbol{x} \in \Gamma$
Robin (**Type 3**)	Head-dependent flux	$\frac{\partial \phi}{\partial n} + a\phi = f(\boldsymbol{x});\ \boldsymbol{x} \in \Gamma$

The Cauchy (Type 4) BC has Dirichlet and Neumann BC independently specified along the same boundary but is rarely used in real world applications. Jazayeri and Werner (2019) document inconsistencies in the literature and found most references to Cauchy (Type 4) BC are misidentified.

Analytical solutions tend to have unbounded (infinite) domains.

A seepage face is an example of a switching BC between Dirichlet and Neumann.

Stress-dependency is of primary concern wherever the model boundaries differ from the natural system boundaries, or the natural boundaries that may extend beyond the boundaries of the model (ASTM 2016). If sensitivity tests indicate stress dependency of a specified head or flux boundary, then the head-dependent flux boundary is suggested.

The practical use of the Robin head dependent flux (Type 3) BC is implemented in MODFLOW as a general head boundary (GHB). The head dependent flux at the boundary cell is defined by a far-field constant head (effectively infinite source of water, hypothetical or real) a set distance away and the material of transfer has an assigned effective conductance (**Fig. S1a**). The head dependent normal flux (Type 3) BC is represented in AnAqSim as an outer domain line element with an estimated effective outside domain conductance and head (**Fig. S1b**).

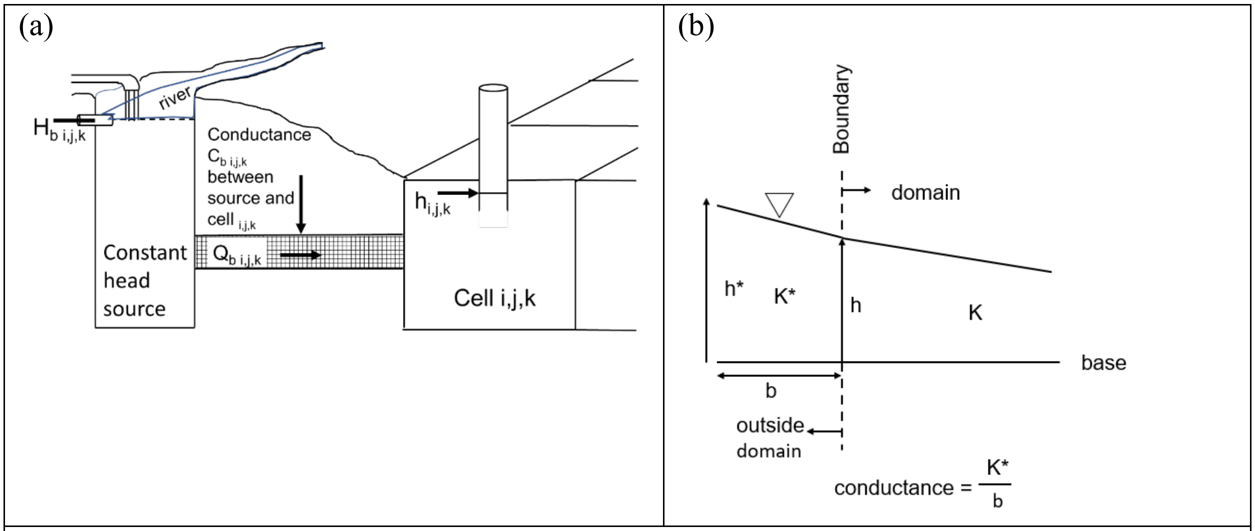

Figure S1. Conceptualization of the Robin (Type 3) head dependent flux BC: (a) presented in the finite difference model MODFLOW for boundary cells, after Reilly (2001) and WaterlooHydrogeologic.com; and (b) presented in the analytic element model AnAqSim as line boundary element, after AnAqSim User Guide.

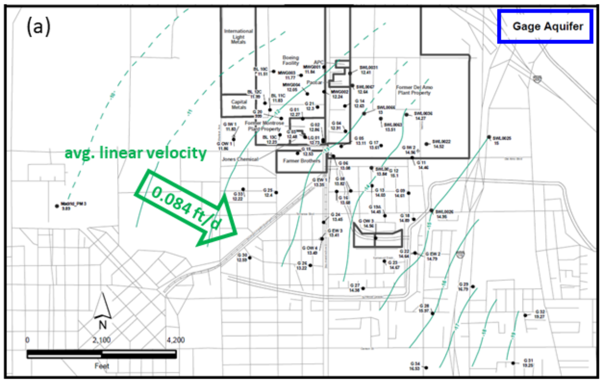

(b)

Head

Observation Group	Name	ALL	HDIFF	HL01	HL02	HL03	HL04	HL05	HL07	HL09	HL13
	Count	320	70	53	88	8	9	77	2	71	12
Observation Summary	Max	-5.02	10.65	-5.02	-9.06	-11.38	-10.56	-9.83	-12.36	-10.16	-10.16
	Min	-29.30	-2.50	-13.50	-13.60	-12.67	-15.44	-16.04	-14.56	-19.27	-29.30
	Range	24.28	13.15	8.48	4.54	1.29	4.88	6.21	2.20	9.11	19.14
Residual Statistics	Max	2.99	3.57	2.99	2.25	0.71	0.63	2.72	0.28	1.61	2.14
	Min	-2.17	-0.88	-0.90	-1.78	-0.01	-0.05	-0.94	-0.55	-2.17	-2.02
	Mean	0.21	0.29	0.50	0.23	0.27	0.28	0.25	-0.14	0.01	-0.36
	Absolute Mean	0.44	0.46	0.61	0.44	0.27	0.29	0.37	0.42	0.33	1.08
	RMS	0.66	0.80	0.87	0.64	0.35	0.33	0.55	0.44	0.54	1.27
	%RMS	2.7%	6.0%	10.3%	14.1%	27.0%	6.8%	8.8%	19.9%	5.9%	6.6%

Gage aquifer

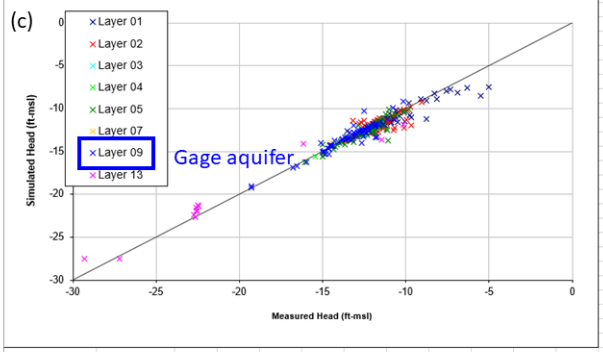

Figure S2. Summary of calibration of the MODFLOW Dual Site model, Layer 9, Gage Aquifer: (a) Simulated water level contours and monitoring wells; (b) calibration statistics for all layers; (c) scatter plot of hydraulic heads all layers. After CH2M Hill 2008.

The pathlines associated with the Dual Site pump-treat-inject system are visualized using the MODFLOW solution for groundwater flow and the MODPATH model for reverse particle tracking from the extraction wells (**Fig. S3**). Attention focused on the Gage aquifer layer that is the target for the injection wells and the model suggests that the injection wells and extraction wells form a closed pathline envelope in the Gage aquifer. The model files come from ICF/Sundance (2019).

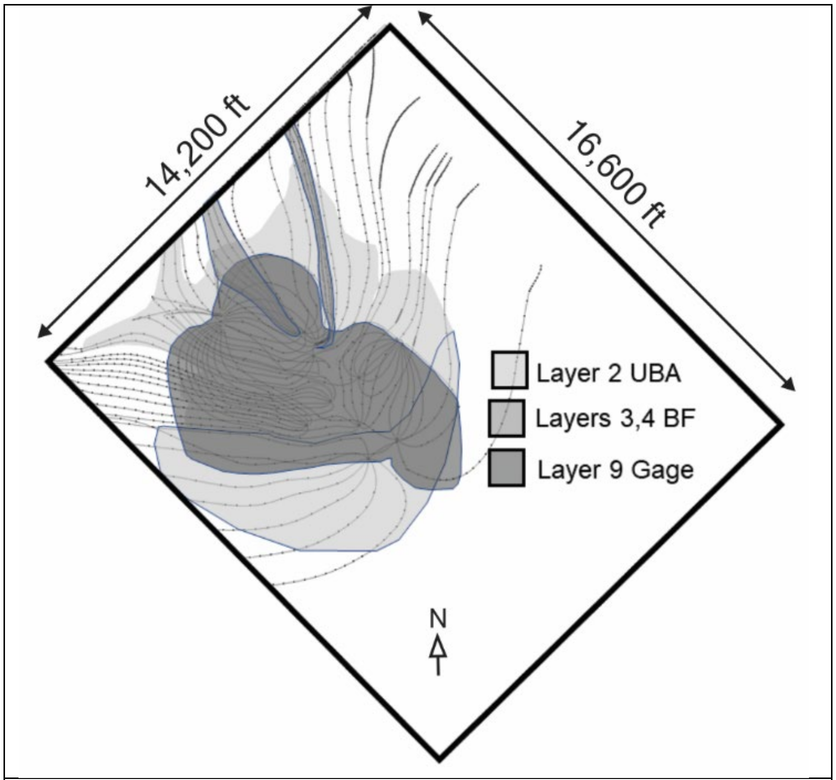

Figure S3. MODFLOW/MODPATH Dual Site capture zones based on tracelines for the extraction wells. The extraction wells are mostly screened in Layer 9 Gage aquifer. Extraction wells are also screen in Layer 2 Upper Bellflower aquifer (UBA) and Layers 3,4 Bellflower aquifer (BF). The injection wells are screened in the Gage aquifer.

Table S2. AnAqSim Dual Site model domain parameterization.

Domain Label	Level Label	Domain Type	Top elevation (ft amsl)	Bottom elevation (ft amsl)	Porosity	K1 horizontal (ft/d)	K2 horizontal (ft/d)	K3 vertical top half (ft/d)	K3 vertical bottom half (ft/d)
Dual Site Mesa L4	4	confined	0.0	-118.2	0.3	23.1	"=K1"	6.2	0.075
Dual Site Pacific L5	5	confined	-118.2	-177.5	0.3	31.5	"=K1"	"=K1*0.1"	"=K1*0.1"
Dual Site Harbor L8	8	confined	-177.5	-323.2	0.3	54.3	"=K1"	2.30E-03	"=K1*0.1"
Dual Site Up WIlm L9	9	confined	-323.3	-1050.3	0.3	20.19	"=K1"	0.0201	2.95E-04
Dual Site Lo Wilm L10	10	confined	-1050.3	-1284.9	0.3	20.19	"=K1"	0.0201	2.95E-04
Subdomain Overburden L1	1	unconfined	0.0	-10.9	0.3	1	"=K1"	"=K1*0.1"	"=K1*0.1"
Subdomain Upper Bellflower aquitard L2	2	confined/unconfined	-10.9	-65.7	0.3	9.64	"=K1"	0.02	0.25
Subdomain Middle Bellflower aquifer L3	3	confined	-65.7	-104.6	0.3	53.6	"=K1"	"=K1*0.1"	"=K1*0.1"
Subdomain Lower Bellflower aquitard L4	4	confined	-104.6	-130.4	0.3	0.085	"=K1"	"=K1*0.1"	"=K1*0.1"
Subdomain Gage aquifer L5	5	confined	-130.4	-192.5	0.3	35.1	"=K1"	"=K1*0.1"	"=K1*0.1"
Subdomain Gage-Lynnwood Aquitard L6	6	confined	-192.5	-233.5	0.3	0.015	"=K1"	"=K1*0.1"	"=K1*0.1"
Subdomain Lynnwood Aquifer upper L7	7	confined	-233.5	-283.5	0.3	57.9	"=K1"	11.6	11.6
Subdomain Lynwood Aquifer lower L8	8	confined	-283.5	-333.8	0.3	57.9	"=K1"	11.6	11.6

Table S3. AnAqSim Dual Site model well parameterization.

Label	Domain	Radius (ft)	Discharge (ft^3/d)
G-IW-3	Dual Site Pacific L5	1	26,308.3
G-IW-7	Dual Site Pacific L5	1	26,308.3
G-IW-1	Dual Site Pacific L5	1	26,308.3
G-IW-4	Subdomain Gage aquifer L5	1	13,956.2
G-IW-2	Subdomain Gage aquifer L5	1	13,956.2
G-IW-5	Subdomain Gage aquifer L5	1	13,956.2
G-IW-6	Subdomain Gage aquifer L5	1	13,956.2
UBA-EW-1	Subdomain Upper Bellflower aquitard L2	1	-4,812.5
BF-EW-5	Subdomain Middle Bellflower aquifer L3	1	-5,197.5
G-EW-1	Subdomain Gage aquifer L5	1	-23,100.0
BF-EW-1	Subdomain Middle Bellflower aquifer L3	1	-8,085.0
MBFB-EW-1	Subdomain Middle Bellflower aquifer L3	1	0.0
UBA-EW-3	Subdomain Upper Bellflower aquitard L2	1	-2,887.5
G-EW-4	Subdomain Gage aquifer L5	1	-20,212.5
BF-EW-3	Subdomain Middle Bellflower aquifer L3	1	-13,860.0
G-EW-3	Subdomain Gage aquifer L5	1	-4,812.5
BF-EW-2	Subdomain Middle Bellflower aquifer L3	1	-13,860.0
BF-EW-4	Subdomain Middle Bellflower aquifer L3	1	-26,372.5
G-EW-2	Subdomain Gage aquifer L5	1	-11,550.0

Table S4. AnAqSim Dual Site model line head dependent normal flux (3rd type) parameterization.

Label	Domain	Parameters per line	Conductance start (1/d)	Conductance end (1/d)	h^* start (ft amsl)	h^* end (ft amsl)
Dual Site_NW_L4	Dual Site Mesa L4	8	6.34E-03	1.92E-03	-9.3	-8.6
Dual Site_SW_L4	Dual Site Mesa L4	8	1.46E-03	1.08E-03	-8.6	-19.6
Dual Site_SE_L4	Dual Site Mesa L4	8	1.46E-03	4.32E-03	-19.6	-21.9
Dual Site_NE_L4	Dual Site Mesa L4	8	8.22E-04	1.21E-03	-21.9	-9.3
Dual Site_NW_L5	Dual Site Pacific L5	8	6.34E-03	1.92E-03	-9.3	-8.6
Dual Site_SW_L5	Dual Site Pacific L5	8	1.46E-03	1.08E-03	-8.6	-19.6
Dual Site_SE_L5	Dual Site Pacific L5	8	1.46E-03	4.32E-03	-19.6	-21.9
Dual Site_NE_L5	Dual Site Pacific L5	8	8.22E-04	1.21E-03	-21.9	-9.3
Dual Site_NW_L8	Dual Site Harbor L8	8	4.66E-02	2.11E-02	-27.3	-9.1
Dual Site_SE_L8	Dual Site Harbor L8	8	2.18E-02	4.09E-02	-24.4	-42.4
Dual Site_NE_L8	Dual Site Harbor L8	8	7.71E-03	8.91E-03	-42.4	-9.3
Dual Site_NW_L9	Dual Site Up WIlm L9	8	4.66E-02	2.11E-02	-27.3	-9.1
Dual Site_SW_L9	Dual Site Up WIlm L9	8	1.60E-02	1.66E-02	-9.1	-24.4
Dual Site_SE_L9	Dual Site Up WIlm L9	8	2.18E-02	4.09E-02	-24.4	-42.4
Dual Site_NE_L9	Dual Site Up WIlm L9	8	7.71E-03	8.91E-03	-42.4	-27.5
Dual Site_NW_L10	Dual Site Lo Wilm L10	8	4.66E-02	2.11E-02	-27.3	-9.1
Dual Site_SW_L10	Dual Site Lo Wilm L10	8	1.60E-02	1.66E-02	-9.1	-24.4
Dual Site_SE_L10	Dual Site Lo Wilm L10	8	2.18E-02	4.09E-02	-24.4	-42.4
Dual Site_NE_L10	Dual Site Lo Wilm L10	8	7.71E-03	8.91E-03	-42.4	-27.5
Dual Site_SW_L8	Dual Site Harbor L8	8	1.60E-02	1.66E-02	-9.1	-24.4

Table S5. AnAqSim LA Basin model domains.

Label	Level	Domain Type	Top Elevation (ft amsl)	Bottom Elevation (ft amsl)	Porosity	K1 horizontal (ft/d)	K2 horizontal (ft/d)	K3 vertical top half (ft/d)	K3 vertical bottom half (ft/d)
Central Basin A L10	10	confined	-217.6	-1,226.90	0.3	30.8	30.8	3.5	2.3
Central Basin A L5	5	confined/unconfined	0	-217.6	0.3	76.6	76.6	17.6	2.86
Central Basin B L10	10	confined	-205.6	-1027.2	0.3	12.84	12.84	1.50E-04	6.70E-05
Central Basin B L5	5	confined/unconfined	0	-205.6	0.3	34.1	34.1	3	3.7
Central Basin E L10	10	confined	-180	-1,152.00	0.3	30.3	30.3	3.348	2.392
Central Basin E L5	5	confined/unconfined	36.1	-180	0.3	31.93	31.93	3.193	3.193
Central Basin W L10	10	confined	-117.5	-863.6	0.3	35.6	35.6	4.9	2.1
Central Basin W L5	5	confined/unconfined	75.4	-117.5	0.3	28.6	28.6	2.86	2.86
Hollywood Basin L10	10	confined	-28.6	-371.5	0.3	14.2	14.2	1.1	2.5
Hollywood Basin L5	5	confined	160	-28.6	0.3	14.45	14.45	1.445	1.445
Dual Site Refined Gage aquifer L5	5	confined	-130.4	-192.5	0.3	35.1	"=K1"	"=K1*0.1"	"=K1*0.1"
Dual Site Refined Gage-Lynnwood Ac	6	confined	-192.5	-233.5	0.3	0.015	"=K1"	"=K1*0.1"	"=K1*0.1"
Dual Site Refined Lower Bellflower a	4	confined	-104.6	-130.4	0.3	0.085	"=K1"	"=K1*0.1"	"=K1*0.1"
Dual Site Refined Lynnwood Aquifer	7	confined	-233.5	-283.5	0.3	57.9	"=K1"	11.6	11.6
Dual Site Refined Lynwood Aquifer lo	8	confined	-283.5	-333.8	0.3	57.9	"=K1"	11.6	11.6
Dual Site Refined Middle Bellflower a	3	confined	-65.7	-104.6	0.3	53.6	"=K1"	"=K1*0.1"	"=K1*0.1"
Dual Site Refined Overburden L1	1	unconfined	0	-10.9	0.3	1	"=K1"	"=K1*0.1"	"=K1*0.1"
Dual Site Refined Upper Bellflower a	2	confined/unconfined	-10.9	-65.7	0.3	9.64	"=K1"	0.02	0.25
LA Forebay E L10	10	confined	-53.8	-711.6	0.3	7.4	7.4	0.764	0.683
LA Forebay E L5	5	confined	106	-53.8	0.3	94.6	94.6	17.91	5.54
LA Forebay W L10	10	confined	-31.8	-462.1	0.3	14.9	14.9	1.68	1.1
LA Forebay W L5	5	confined	127.3	-31.8	0.3	14.5	14.5	1.45	1.45
Montebello Forebay L10	10	confined	-98.9	-857.3	0.3	55.8	55.8	7.1	2.6
Montebello Forebay L5	5	confined	46.4	-98.9	0.3	130.2	130.2	22.7	6.2
N-I Fault Central Basin A L10	10	confined	-217.6	-1,226.90	0.3	0.1	0.1	0.1	0.1
N-I Fault Central Basin A L5	5	confined/unconfined	0	-217.6	0.3	0.1	0.1	0.1	0.1
N-I Fault Central Basin B L10	10	confined	-205.6	-1027.2	0.3	0.1	0.1	0.1	0.1
N-I Fault Central Basin B L5	5	confined/unconfined	0	-205.6	0.3	0.1	0.1	0.1	0.1
N-I Fault Central Basin W L10	10	confined	-117.5	-863.6	0.3	0.1	0.1	0.1	0.1
N-I Fault Central Basin W L5	5	confined/unconfined	75.4	-117.5	0.3	0.1	0.1	0.1	0.1
N-I Fault West Coast Basin NW L10	10	confined	-496.4	-1060.6	0.3	0.1	0.1	0.1	0.1
N-I Fault West Coast Basin NW L4	4	confined/unconfined	0	-59.6	0.3	0.1	0.1	0.1	0.1
N-I Fault West Coast Basin NW L5	5	confined/unconfined	-59.6	-187.9	0.3	0.1	0.1	0.1	0.1
N-I Fault West Coast Basin NW L8	8	confined	-187.9	-496.4	0.3	0.1	0.1	0.1	0.1
Orange County Central L10	10	confined	-180	-1,152.00	0.3	30.3	30.3	3.348	3.348
Orange County Central L5	5	confined/unconfined	36.1	-180	0.3	31.93	31.93	"=K1*0.1"	"=K1*0.1"
Orange County North L10	10	confined	-13.6	-723.6	0.3	6.4	6.4	0.616	0.616
Orange County North L5	5	confined	162.2	-13.6	0.3	3.08	3.08	"=K1*0.1"	"=K1*0.1"
Orange County South L10	10	confined	-205.6	-1027.2	0.3	12.84	12.84	1.50E-04	1.50E-04
Orange County South L5	5	confined	0	-205.6	0.3	34.1	34.1	3	3.7
San Pedro Bay N L10	10	confined	-263.7	-1022.7	0.3	45	45	5.7	3
San Pedro Bay N L5	5	confined	0	-263.7	0.3	32.2	32.2	4.7	1.6
San Pedro Bay S L10	10	confined	-243.2	-1024.5	0.3	34.2	34.2	3.8	3
San Pedro Bay S L5	5	confined	0	-243.2	0.3	6.7	6.7	0.67	0.67
Santa Monica Basin L10	10	confined	-80.5	-340.3	0.3	14.7	14.7	0.78	2.95
Santa Monica Basin L5	5	confined/unconfined	0	-80.5	0.3	7.8	7.8	0.78	0.78
Santa Monica Bay L10	10	confined	-245.7	-553.2	0.3	51.7	51.7	6.9	3
Santa Monica Bay L5	5	confined	0	-245.7	0.3	38.8	38.8	3.88	3.88
Santa Monica Bay N L10	10	confined	-250.7	-456.5	0.3	45.5	45.5	5.5	3
Santa Monica Bay N L5	5	confined	0	-250.7	0.3	12.5	12.5	1,25	1.25
Dual Site Harbor L8	7	confined	-177.5	-323.2	0.3	54.3	"=K1"	2.30E-03	"=K1*0.1"
Dual Site Lo Wilm L10	10	confined	-1050.3	-1284.9	0.3	20.19	"=K1"	0.0201	2.95E-04
Dual Site Mesa L4	4	confined/unconfined	0	-118.2	0.3	23.1	"=K1"	6.2	0.075
Dual Site Pacific L5	5	confined	-118.2	-177.5	0.3	31.5	"=K1"	"=K1*0.1"	"=K1*0.1"
Dual Site Up WIlm L9	9	confined	-323.2	-1050.3	0.3	20.19	"=K1"	0.0201	2.95E-04
West Coast Basin E L10	10	confined	-292.6	-699.9	0.3	21.76	21.76	0.0606	2.51E-03
West Coast Basin E L4	4	confined/unconfined	0	-75	0.3	21	21	0.027	0.133
West Coast Basin E L5	5	confined/unconfined	-75	-170.6	0.3	21	21	0.027	0.133
West Coast Basin E L8	8	confined	-170.6	-292.6	0.3	21.76	21.76	2.51E-03	2.51E-03
West Coast Basin NE L10	10	confined	-494.2	-1231.9	0.3	21.76	21.76	0.0606	2.51E-03
West Coast Basin NE L4	4	confined/unconfined	0	-115.8	0.3	21	21	0.027	0.133
West Coast Basin NE L5	5	confined/unconfined	-115.8	-227	0.3	23.3	23.3	0.012	0.012
West Coast Basin NE L8	8	confined	-227	-494.2	0.3	21.76	21.76	2.51E-03	2.51E-03
West Coast Basin NW L10	10	confined	-496.4	-1060.6	0.3	20.19	20.19	0.0201	2.95E-04
West Coast Basin NW L4	4	confined/unconfined	0	-59.6	0.3	23.31	23.31	0.012	0.012
West Coast Basin NW L5	5	confined/unconfined	-59.6	-187.9	0.3	23.31	23.31	0.012	0.012
West Coast Basin NW L8	8	confined	-187.9	-496.4	0.3	20.19	20.19	0.0201	2.95E-04
West Coast Basin SE L10	10	confined	-374.3	-847.6	0.3	21.76	21.76	0.0606	2.51E-03
West Coast Basin SE L4	4	confined/unconfined	0	-70	0.3	21	21	0.027	0.133
West Coast Basin SE L5	5	confined/unconfined	-70	-150.5	0.3	21	21	0.027	0.133
West Coast Basin SE L8	8	confined	-150	-374.3	0.3	21.76	21.76	2.51E-03	2.51E-03
West Coast Basin SW L10	10	confined	-569	-1032.5	0.3	20.19	20.19	0.0201	2.95E-04
West Coast Basin SW L4	4	confined/unconfined	0	-37.1	0.3	23.31	23.31	0.012	0.012
West Coast Basin SW L5	5	confined/unconfined	-37.1	-183	0.3	23.31	23.31	8.11E-03	1.326
West Coast Basin SW L8	8	confined	-183	-569	0.3	23.31	23.31	0.0201	2.95E-04
West Coast Basin W L10	10	confined	-500.9	-1029	0.3	20.19	20.19	0.0201	2.95E-04
West Coast Basin W L4	4	confined/unconfined	0	-85.2	0.3	23.31	23.31	0.012	0.012
West Coast Basin W L8	8	confined	-223.8	-500.9	0.3	20.19	20.19	0.0201	2.95E-04
West Cost Basin W L5	5	confined/unconfined	-85.2	-223.8	0.3	23.31	23.31	0.012	0.012
Whittier Basin L10	10	confined	-13.6	-723.6	0.3	6.4	6.4	0.616	0.69
Whittier Basin L5	5	confined	162.2	-13.6	0.3	3.08	3.08	"=K1*0.1"	"=K1*0.1"

Table S6. AnAqSim LA Basin spatially variable area sources/sinks by domain

Domain	Condition top	Top flux (ft/d) or head (ft amsl)	Node spacing (ft)
Hollywood Basin L5	Flux	6.12E-04	4000
Montebello Forebay L5	Flux	-3.88E-03	2000
Orange County Central L5	Flux	-4.22E-03	4000
Orange County North L5	Flux	1.28E-04	4000
Orange County South L5	Flux	-8.61E-06	4000
San Pedro Bay N L5	Head_dependent_flux	1.5	4000
San Pedro Bay S L5	Head_dependent_flux	1.5	4000
Santa Monica Basin L5	Flux	1.75E-04	4000
Santa Monica Bay L5	Head_dependent_flux	2.7	4000
Santa Monica Bay N L5	Head_dependent_flux	2.7	4000
West Coast Basin E L4	Flux	-8.00E-04	4000
West Coast Basin NE L4	Flux	0	4000
West Coast Basin NW L4	Flux	0	4000
West Coast Basin SE L4	Flux	0	4000
West Coast Basin SW L4	Flux	-8.00E-04	4000
West Coast Basin W L4	Flux	-8.00E-04	4000
Whittier Basin L5	Flux	2.74E-04	4000

Table S7. AnAqSim LA Basin spatially variable area sources/sinks by polygon.

Label	Nesting Level	Condition Top	Top flux (ft/d) or head (ft amsl)	Node spacing (ft)
LA Forebay NonPressure WRD	1	Flux	-3.04E-03	4000
LA Forebay NonPressure nonWRD	1	Flux	4.90E-04	4000
Central Basin PressureArea nonWRD	1	Flux	3.81E-04	4000
Montebello Forebay Spreading W	2	Flux	2.00E-01	1000
Montebello Forebay Spreading E	2	Flux	2.00E-01	1000
Dual Site	1	Flux	2.90E-04	400
Dual Site refined	2	Flux	2.90E-04	400
Central Basin PressureArea WRD	1	Flux	-3.27E-03	4000

The Los Angeles Basin including the Central, West Coast, and Orange County Basins, as represented in the single level steady GFLOW groundwater model, has outer boundary conditions having mostly geohydrological correspondence: no-flow boundaries associated with rock hills; specified head boundaries associated with the ocean shorelines; specified flux focused through the LA Narrows and Whittier Narrows. The layout of elements is shown in **Fig. S4**.

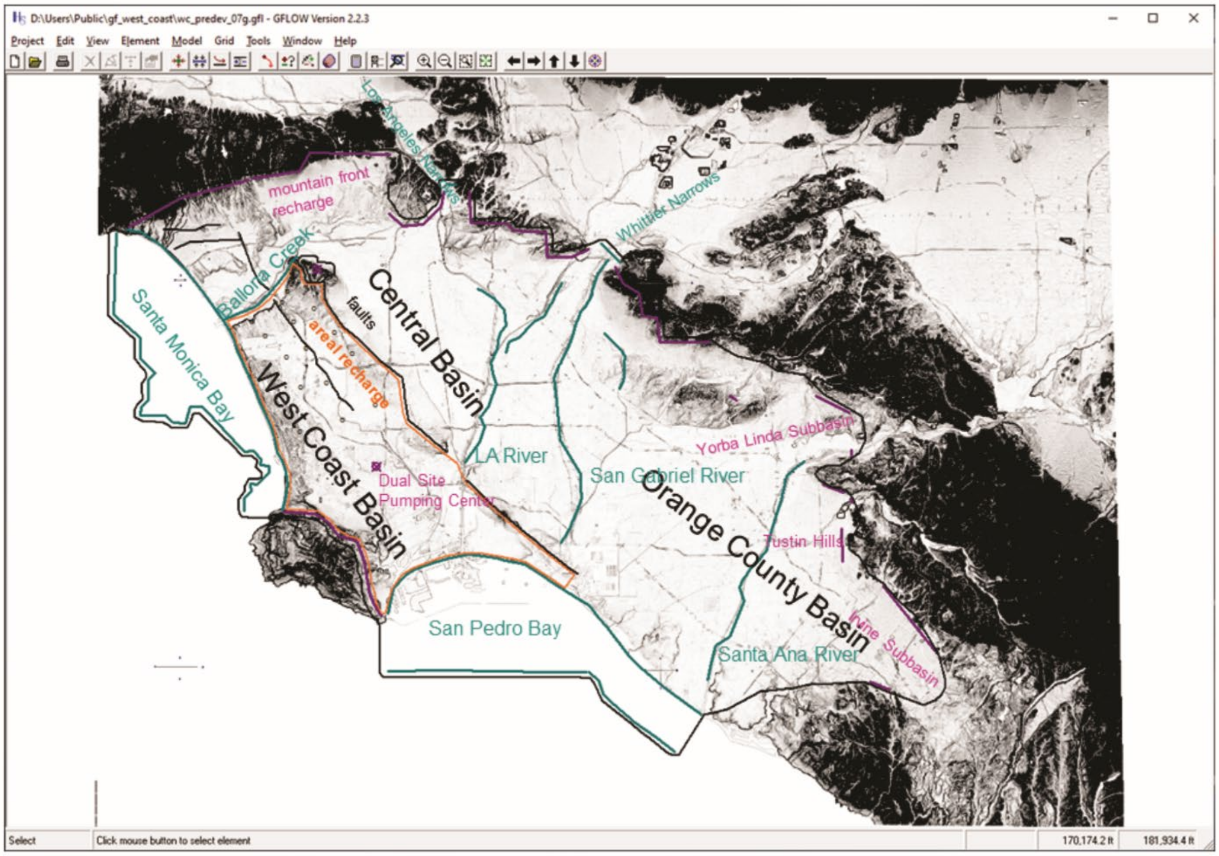

Figure S4. GFLOW layout of the "predevelopment" Los Angeles Coastal Plain including Los Angeles County and Orange County, California USA. The basemap is the USGS 1 arc second DEM. The analytic elements include constant head line-sinks (h=0.0 ft) for the Santa Monica Bay and the San Pedro Bay; specified head line-sinks (linearly variable) for the LA River, San Gabriel River, Santa Ana River, Ballona Creek, LA Narrows, Whittier Narrows; specified discharge line-sinks for mountain front recharge associated with Yorba Linda subbasin, Tustin Hills, Irvine subbasin, La Habra subbasin, Santa Monica Hills, Hollywood Hills, Elysian Hills, Repeto Hills, Merced Hills ; horizontal barrier line elements (no-flow) surrounding the basin; horizontal barriers line elements (leakage) for the Newport-Inglewood fault zones; area elements for recharge in the West Coast basin. The Dual Site Montrose-Del Amo Superfund site near Torrance is shown. For "predevelopment" conditions no pumping wells are explicitly introduced.

The calibration of GFLOW for “predevelopment” conditions are shown in **Fig. S5** and **S6**. In **Fig. S5**, the basemap is the map of head contours reported by Mendenhall (1905). The shaded areas were artesian at that time prior to over pumping. GFLOW test points were associated with the 10, 20, 30 foot contours in the West Coast Basin. GFLOW shows triangles centered on test points -- red triangles with tip pointing down show model prediction less than observed, green triangles with tip pointing up show model prediction greater than observed. Ideally the distribution of triangles in space and in size should be random to minimize bias.

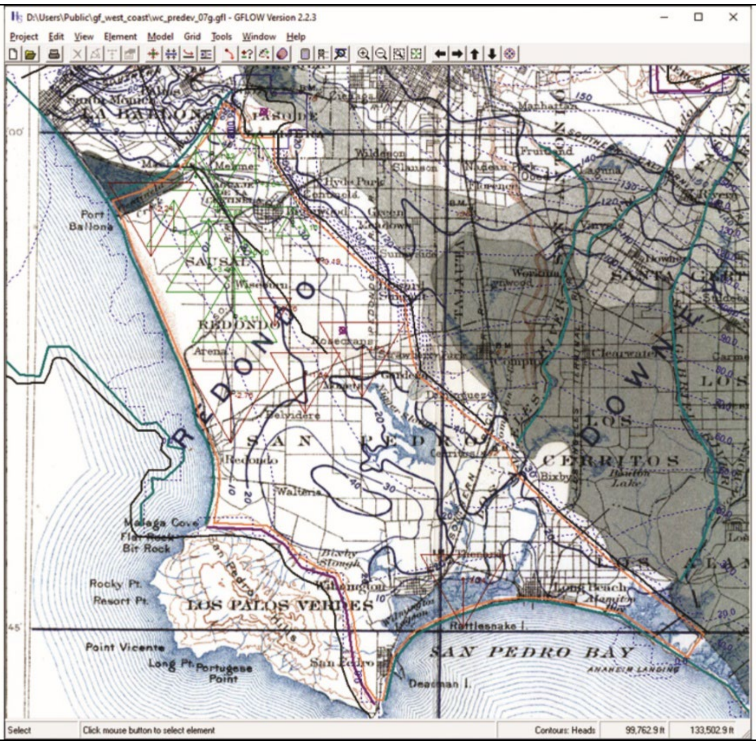

Figure S5. The GFLOW head contours for “predevelopment” conditions are shown as dashed lines with 10 ft intervals. The GFLOW basemap is the map of observations and head contours reported by Mendenhall (1905). GFLOW test points were associated with the 10, 20, and 30 foot contours in the West Coast Basin. GFLOW shows triangles centered on test points -- red triangles with tip pointing down show model prediction less than observed, green triangles with tip pointing up show model prediction greater than observed.

In **Figure S6**, the GFLOW calibration statistics are shown for the test points. Parameterization is based on MODFLOW (Reichard et al. 2003) including effective horizontal hydraulic conductivity for West Coast Basin, and mountain front recharge discharge specified linesinks. The simple conceptual model, averaged steady flow, single layer with horizontal base elevation, single hydraulic conductivity and West Coast basin calibrated areal recharge gives minimized mean residual 0.0 ft and normalized RMSE 28.9%. The dashed lines represent the eastern artificial boundary of the USGS Paulinski et al. (2021) MODFLOW-USG. Notice that the head contours are perpendicular to this line under pre-development conditions suggesting justification of an artificial no-flow BC. Superimposing a flux condition to the OC line would reflect the influence of well extractions and barrier well injections.

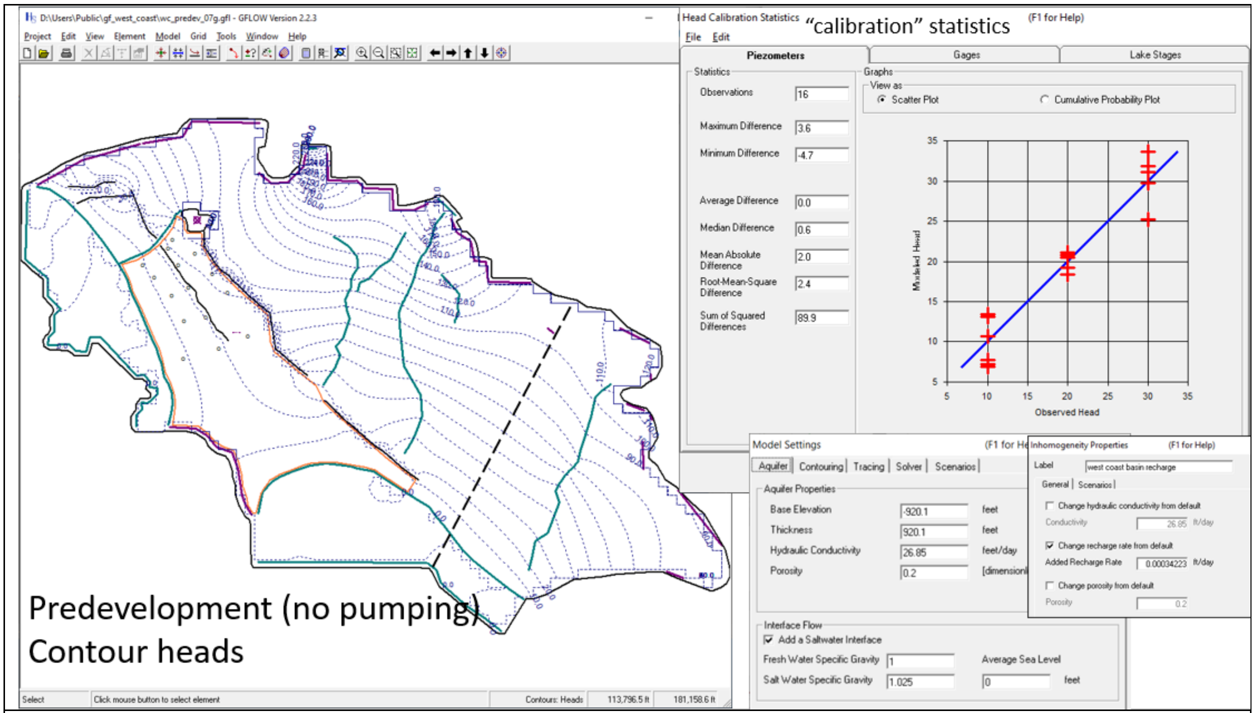

Figure S6. GFLOW calibration to “predevelopment” West Coast basin test points based on Mendenhall (1905) head contours (10 ft, 20 ft, 30 ft). Parameterization based on MODFLOW (Reichard et al. 2003) including effective horizontal hydraulic conductivity for West Coast Basin, and mountain front recharge discharge specified linesinks. The statistics include the minimized mean residual 0.0 ft and normalized RMSE 28.9%. The dashed line represents the location of the Paulinski et al. (2021) Orange County MODFLOW model boundary.

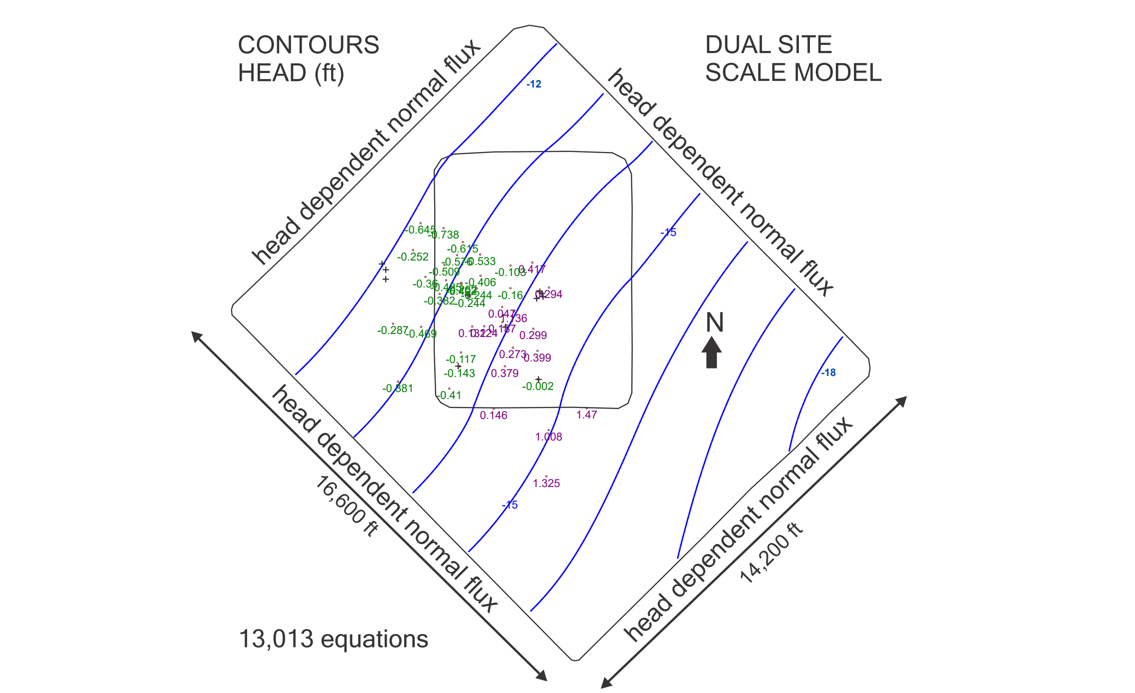

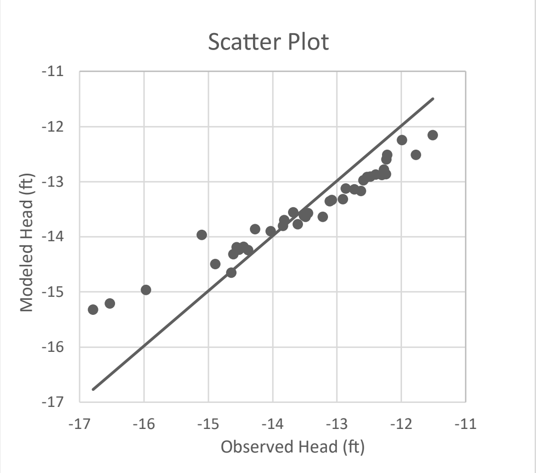

Head, Observation Group	Name	Level 5 Gage Aquifer
	Count	39
Observation Summary	Max (ft)	-11.51
	Min (ft)	-16.79
	Range (ft)	5.28
Residual Statistics	Max (ft)	1.47
	Min (ft)	-0.74
	Mean (ft)	-0.028
	RMSE (ft)	0.530
	Norm. RMSE (%)	10.04

Figure S7. Calibration of the AnAqSim model for the Dual Site scale. The bias in the Dual Site scale AnAqSim modeled heads in the Gage aquifer (Level 5) (green test points values being model too low; red being model too high) is likely due to the simplification of domain levels with horizontal bottoms.

(a)

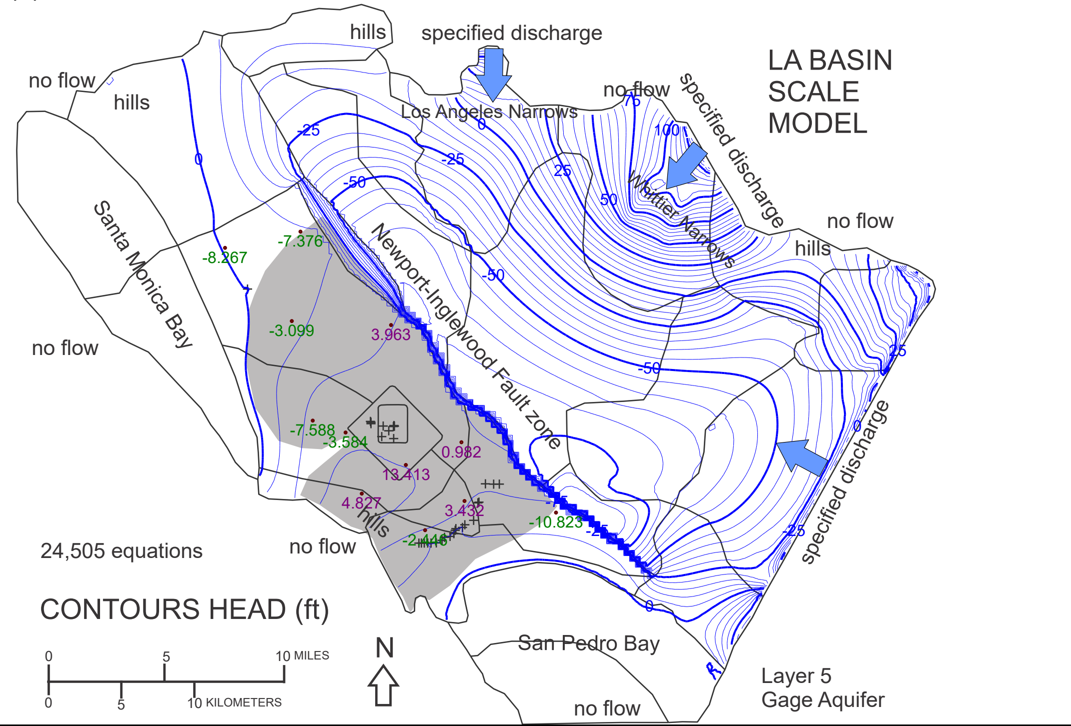

(b)

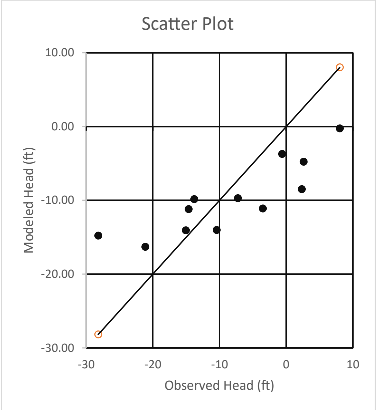

(c)

Head, Observation Group	Name	Level 5 Gage Aquifer
	Count	12
Observation Summary	Max (ft)	8.01
	Min (ft)	-28.21
	Range (ft)	36.22
Residual Statistics	Max (ft)	13.41
	Min (ft)	-10.82
	Mean (ft)	-1.38
	RMSE (ft)	6.82
	Norm. RMSE (%)	18.82

Figure S8. Calibration of the AnAqSim model for the Los Angeles Basin scale. The area of drawdown influence for when the Dual Site wells are turned on is superimposed in grey shade.

(a)

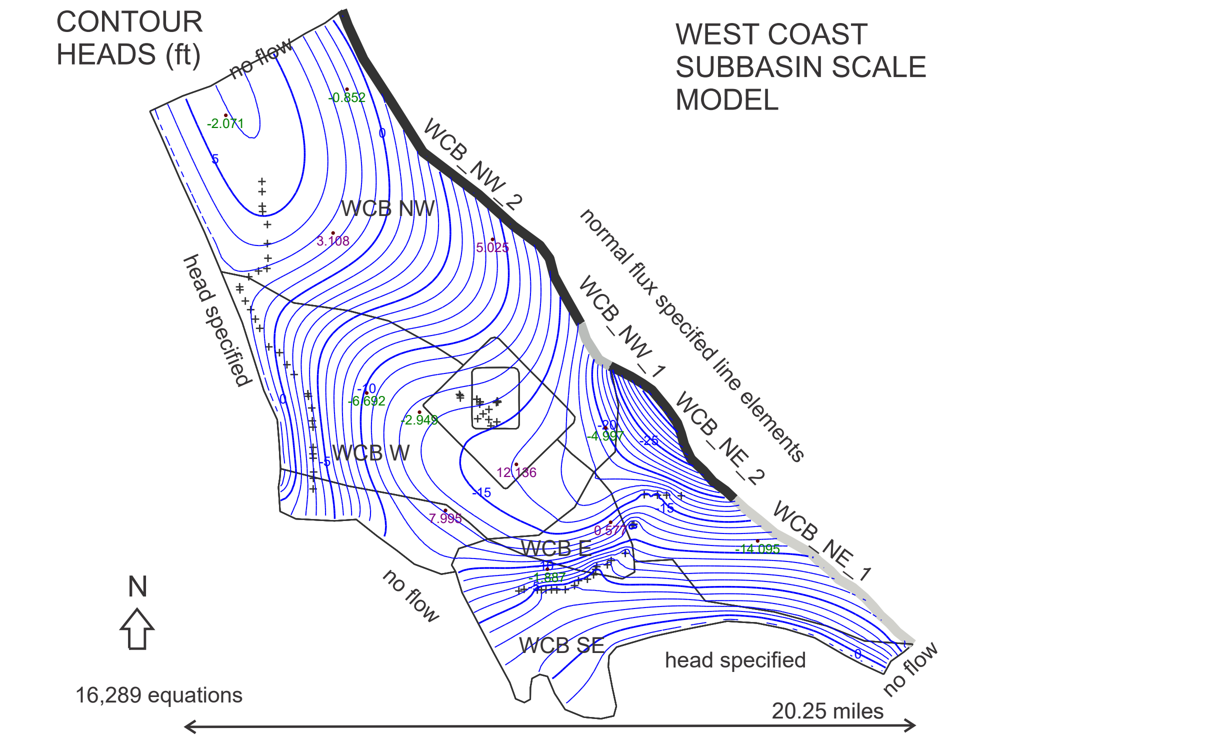

(b)

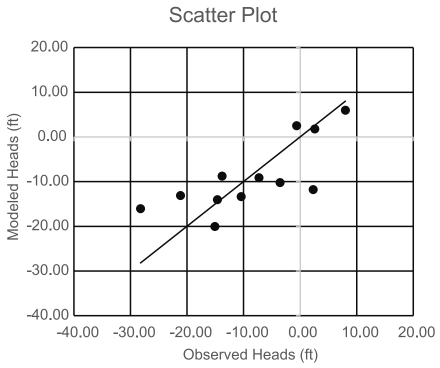

(c)

Head, Observation Group	Name	Level 5 Gage Aquifer
	Count	12
Observation Summary	Max (ft)	8.01
	Min (ft)	-28.21
	Range (ft)	36.22
Residual Statistics	Max (ft)	12.14
	Min (ft)	-14.10
	Mean (ft)	-0.392
	RMSE (ft)	6.949
	Norm. RMSE (%)	19.18

Figure S9. Calibration of the AnAqSim model for the West Coast subbasin scale. The residuals at the test points are fairly randomly distributed.

The contours of hydraulic heads for spring and fall of 2006 are mapped in the West Coast Basin and Central Basin Los Angeles County (**Fig. S10**). The maps reveal a difference in seasonal extractions, the performance of the Newport Inglewood uplift as a hydraulic barrier, and the potential for influence on the groundwater flow on the Dual Site MODFLOW area of simulation. The drawdown in the highlighted Long Beach No. 6 well occurs in the Silverado and Lynwood aquifers (-380 to -500 ft below ground surface).

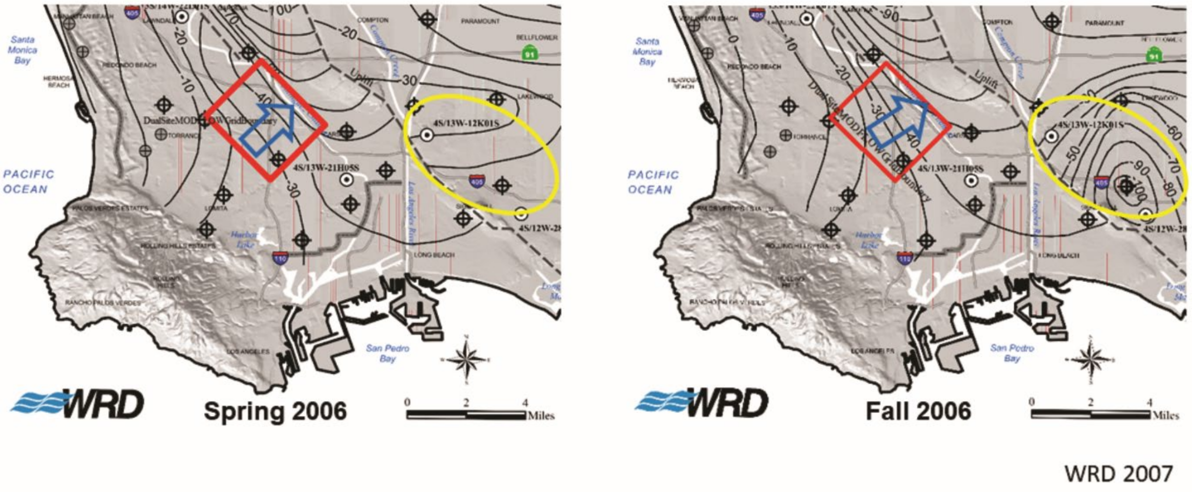

Figure S10. Contours of averaged hydraulic heads in productive aquifers for Spring and Fall of 2006 in the West Coast Basin and Central Basin, Los Angeles County (WRD, 2007). The outer boundary of the MODFLOW grid for the Dual Site model is shown in red. The highlighted yellow area shows the influence of the fall extractions and the Newport Inglewood uplift as a hydraulic barrier.

MODEL FILES and Datasets

The supporting model files and datasets are available for download at data.gov, go to DATA, go to Organizations, search for U.S. Environmental Protection Agency to arrive at the EPA ScienceHub. Search for Dual Site for metadata and files.

AnAqSim project files and associate solutions will run in unlicensed (free) mode for inspection. A license will be required to create new solutions for models that have more than 3 levels and more than 2000 equations.

GFLOW project files and basemaps have no restrictions.

MODFLOW files were provided courtesy of the Dual Site Superfund EPA Region 9, Remedial Project Manager.

Supplementary References

AnAqSim software and user guide are available for download (educational version is free) at https://www.fittsgeosolutions.com

ASTM. 2016. Standard guide for defining boundary conditions in groundwater flow modeling, D5609-16, https://doi.org/10.1520/d5609-16

CH2M Hill. 2008. Model Development and Remedial Wellfield Optimization Report: Dual Site Groundwater Operable Unit Remedial Design, Montrose Chemical and Del Amo Superfund Sites, prepared for US EPA Region 9, San Francisco, https://semspub.epa.gov/work/09/2192863.pdf

GFLOW software and documentation is available for free download at https://www.epa.gov/ceam/gflow-groundwater-flow-analytic-element-model

ICF/Sundance. 2019. Montrose-Del Amo Three-dimensional visualization and analysis (3DVA), Technical Memorandum under EPA Contract EP-W-14-001, contact the author.

Jazayeri, A., A.D. Werner. 2019. Boundary condition nomenclature confusion in groundwater flow modeling, Ground Water, 57(5):664-668, https://doi.org/10.1111/gwat.12893

Mendenhall, W.C. 1905. Development of underground water in the Western Coastal Plain Region of Southern California, U.S. Geological Survey Water Supply and Irrigation Paper No. 139, 117 pp., with Plates, https://pubs.er.usgs.gov/publication/wsp139

Paulinski, S., ed.. 2021. Development of a groundwater-simulation model in the Los Angeles Coastal Plain, Los Angeles County, California: U.S. Geological Survey Scientific Investigations Report 2021-5088, 489 p., https://doi.org/10.3133/sir20215088

Reichard E.G., M. Land, S.M. Crawford, T. Johnson, R.R. Everett, T.V. Kulshan, D.J. Ponti, K.J. Halford, T.A. Johnson, K.S. Paybins, T. Nishikawa. 2003. Geohydrology, geochemistry, and ground-water simulation-optimization of the Central and West Coast Basins, Los Angeles, County, California, U.S. Geological Survey Water-Resources Investigations Report 03-4065, https://doi.org/10.3133/wri034065

Reilly, T.E. 2001. System and boundary conceptualization in ground-water flow simulation, Techniques of Water-Resources Investigations of the U.S. Geological Survey, Book 3, Applications of Hydraulics, Chapter B8, https://pubs.er.usgs.gov/publication/twri03B8

WRD. 2007. Regional Groundwater Monitoring Report Water Year 2005-2006, Central and West Coast Basins, Los Angeles County, California, Water Replenishment District of Southern California, April 2007, https://www.wrd.org/regional-groundwater-monitoring-report
